# Multidimensional Analysis Reveals the Flavor Quality Formation Mechanism During the Primary Pile Fermentation of Dark Tea

**DOI:** 10.3390/foods15020212

**Published:** 2026-01-07

**Authors:** Dunchao Wu, Yufei He, Juanshu Wen, Hongfa Zheng, Xi Zhao, Penghui Yu, Ni Zhong, Li Niu, Shi Li, Yong Lin, Hao Huang, Zhonghua Liu

**Affiliations:** 1 Key Laboratory of Tea Science of Ministry of Education, Hunan Agricultural University, Changsha 410128, China; dunchao_wu@163.com (D.W.);; 2 Tea Research Institute, Hunan Academy of Agricultural Sciences, Changsha 410125, China; 3 Yuelushan Laboratory, Changsha 410128, China; 4 National Research Center of Engineering Technology for Utilization of Functional Ingredients from Botanicals, Hunan Agricultural University, Changsha 410128, China

**Keywords:** pile fermentation, multi-enzymes synergistic effect, volatile compounds, GC-MS

## Abstract

Pile fermentation is a crucial process for developing the characteristic mellow taste and aged aroma of dark tea, yet the internal quality transformation mechanism of this process is still unclear. This study employed a high-sensitivity analytical platform based on gas chromatography–mass spectrometry (GC-MS) to systematically investigate the dynamic interplay between key chemical components, enzyme activities, and volatile compounds during the pile fermentation of primary dark tea. Our findings revealed a significant decrease in ester-type catechins, crude protein, and protopectin, alongside a notable accumulation of non-ester-type catechins, gallic acid, and soluble components. The multi-enzyme system—comprising PPO/POD, pectinase/cellulase, and protease—cooperatively drove the oxidation of phenols, cell wall degradation, and the release of aromatic precursors. This was complemented by GC-MS analysis, which identified and quantified 103 volatile compounds across nine chemical classes. The total content of volatile compounds increased significantly, with alcohols, esters, and aldehydes/ketones being the dominant groups. Floral and fruity compounds such as linalool and geraniol accumulated continuously, while esters exhibited an initial increase followed by a decrease. Notably, carotenoid degradation products, including β-ionone, were significantly enriched during the later stages. This study revealed a “oxidation–hydrolysis–reconstruction” metabolic mechanism co-driven by microbial activity and a multi-enzyme system, providing a theoretical foundation for the precise regulation of pile fermentation and targeted quality improvement of primary dark tea.

## 1. Introduction

Dark tea is one of the six major tea categories in China, produced through a distinctive solid-state fermentation process [1]. This tea type is renowned for its mellow, rich taste and characteristic aged aroma [2], and has been reported to exhibit potential health benefits such as regulating blood glucose, alleviating hyperlipidemia, and improving gastrointestinal function [3,4]. The piling fermentation of primary dark tea constitutes a core process, which develops the characteristic quality and style of Chinese dark teas, such as Pu-erh tea and Fu brick tea [5]. During piling fermentation, a series of complex biochemical reactions occur under the coordinated influence of temperature, humidity, microorganisms, and endogenous enzymes [3,6]. This stage plays a decisive role in modulating the transformation of tea constituents, improving infusion taste, and promoting the formation of aroma compounds [3,7].

The quality characteristics of primary dark tea are determined jointly by its taste compounds (non-volatile components) and aroma compounds (volatile components). The dynamic changes in tea constituents during the piling fermentation process constitute the material basis for quality formation, involving complex transformations across multiple categories such as polyphenols, amino acids, alkaloids, and carbohydrates [2,8]. In particular, oxidative polymerization reactions catalyzed by enzymes such as polyphenol oxidase drive the degradation of tea polyphenolic compounds (e.g., EGCG, EC, and EGC) into theaflavins, thearubigins, and theabrownins. These transformations not only profoundly influence the color of the tea infusion but also significantly enhance its mellowness and richness of taste [2,7]. Additionally, gallic acid, a key intermediate product in polyphenol degradation, reflects the intensity and direction of oxidation reactions [9]. Under enzymatic hydrolysis, macromolecules such as proteins and pectins are broken down, resulting in an increase in free amino acids, soluble sugars, and soluble pectins. These compounds not only contribute to the umami taste of the tea infusion but also provide nitrogen sources for microbial metabolism. This series of reactions is mainly regulated by the activity changes in endogenous enzymes—such as polyphenol oxidase (PPO) and peroxidase (POD)—as well as exogenous enzymes (e.g., cellulase, pectinase) produced by microbial metabolism, which collectively determine the pathways of substance transformation [10,11,12].

Furthermore, aroma precursor substances including fatty acids, amino acids, geraniol, and carotenoids undergo enzymatic oxidation, degradation, and transformation. This process leads to the formation and gradual accumulation of key volatile compounds, such as alcohols, aldehydes, esters, and heterocyclic compounds, which collectively establish the characteristic aged and complex aroma profile of primary dark tea [4,12,13]. In recent years, the development of GC-MS has provided a powerful tool for the qualitative and quantitative analysis of volatile aroma components during pile fermentation [14]. However, most existing studies remain focused on the static chemical comparison of the final products. This leaves the systematic investigation into the dynamic interrelationships, such as those among functional compound transformation, enzyme activity variation, and aroma evolution during piling fermentation, relatively understudied.

In this study, samples collected during the piling fermentation process of primary dark tea were analyzed through physicochemical measurements, the quantitative determination of major functional components (e.g., catechins), and dynamic monitoring of enzyme activities. Coupled with GC–MS analysis, the evolution of aroma characteristics was further elucidated. This systematic investigation revealed the variation patterns of key constituents, the role of the enzymatic system in substance transformation, and their intrinsic relationships with aroma characteristics during pile fermentation. Through multidimensional data analysis, a biochemical model of quality formation during the pile fermentation of primary dark tea was established, providing a theoretical basis for scientifically regulating the fermentation process, optimizing flavor profiles, and achieving precise control over tea quality.

## 2. Materials and Methods

### 2.1. Chemicals and Reagents

Deionized water was produced using a Milli-Q water purification system (Millipore, Billerica, MA, USA). n-Alkanes (C3-C7 and C8-C40, standard grade for GC) and *N*,*N*-dimethylformamide (analytical grade) were obtained from Sinopharm Chemical Reagent Co., Ltd. (Shanghai, China). Methanol (LC grade) and acetic acid (LC grade) were purchased from Tedia (Fairfield, OH, USA). Sodium chloride (analytical grade) was purchased from Tedia. All reference standards, including epigallocatechin gallate (EGCG), epicatechin gallate (ECG), epigallocatechin (EGC), gallocatechin gallate (GCG), epicatechin (EC), gallocatechin (GC), DL-catechin (DL-C), gallic acid, caffeine, and theobromine, had a certified purity of ≥98%. They were purchased from Aladdin (Shanghai, China) and J&K Scientific Ltd. (Beijing, China).

All biochemical analyses were performed using commercial assay kits: soluble protein (BCA Protein Assay Kit, YX-W-C202), protopectin (Protopectin Content Assay Kit, YX-C-C113), soluble pectin (WSP Assay Kit, YX-W-WSP), pectinase activity (Kit, YX-W-B621), cellulase activity (CL Assay Kit, YX-W-B610), protease activity (ACP Assay Kit, YX-W-B300), peroxidase activity (POD Assay Kit, YX-W-A502), and polyphenol oxidase activity (PPO Assay Kit, YX-W-A404), all from Sinobestbio (Shanghai, China).

### 2.2. Sample Preparation

Fresh leaves of *Camellia sinensis* cultivar “Baojing Huangjincha No. 1” (one bud with four to five leaves) harvested by machine in June were used as raw material. After spreading for 8 h, the leaves were moistened and subjected to fixation for 3 min at 280 °C, followed by rolling for 30 min. The piling fermentation was then carried out at ambient temperature for 48 h [15]. Using a five-point sampling method, samples were systematically collected from a single pile at 0, 8, 16, 24, 32, 40, and 48 h during pile fermentation. The collected samples were divided into two portions and processed as follows: one portion was flash-frozen in liquid nitrogen and stored at −80 °C for enzyme activity analysis; the second portion was vacuum freeze-dried for the determination of quality-related chemical compositions. Following this treatment, all samples were individually packaged in light-proof, moisture barrier aluminum foil bags and hermetically heat-sealed, ensuring an airtight environment to maintain their chemical integrity until analysis.

### 2.3. Physicochemical Component Analysis

Water extract, tea polyphenols, and free amino acids were measured following Chinese National Standards GB/T 8305-2013 [16], GB/T 8313-2018 [17], and GB/T 8314-2013 [18], respectively; the total flavonoid content was determined using the aluminum chloride colorimetric method [19]. This method mainly reflects the content of flavonoids capable of forming complexes with aluminum ions. The complete detailed procedures are provided in Supplementary File S1.

Catechins, alkaloids, and other related components were determined using high-performance liquid chromatography (HPLC) under the following conditions: a C18 column (4.6 × 150 mm) with a mobile phase consisting of water (phase A) and a mixture of *N*,*N*-dimethylformamide/methanol/acetic acid = 39.5:2:1.5 (phase B), under a gradient elution program as follows: 0–13 min, 14–23% B; 13–28 min, 23–36% B; 28–31 min, 36% B; 31–34 min, 36–14% B; 34–43 min, 14% B. The flow rate was maintained at 1.0 mL/min, the detection wavelength was set at 278 nm, the column temperature at 35 °C, and the injection volume was 10 μL.

The qualitative and quantitative analytical procedures for catechins, alkaloids, and related compounds were conducted as follows. Qualitative analysis: Under the established chromatographic conditions, the mixed standard solution and the corresponding compounds in tea samples achieved satisfactory baseline separation. Compound identification was accomplished by directly comparing the retention times of analytes in the samples with those of the corresponding reference standards analyzed under identical conditions. Representative chromatograms and the corresponding retention times are provided in Figure S1. Quantitative analysis: The mixed standard solution was serially diluted in equal proportions to obtain a series of standard solutions with different concentrations, which were analyzed under the same chromatographic conditions as the samples. Calibration curves were constructed for each target compound by linear regression of peak area (x) versus the known concentration (y). The content of each target compound in the test samples was then calculated by substituting its measured peak area into the corresponding calibration curve equation. Calibration curves are provided in Table S1.

Crude and soluble protein contents were determined using the bicinchoninic acid (BCA) method [20]. Protopectin and soluble pectin contents were analyzed by the imidazole colorimetric method [21]. The cellulose and soluble sugars were determined by the anthrone–sulfuric acid method. The complete detailed procedures are provided in Supplementary File S1.

### 2.4. Enzyme Activity Determination

The activities of polyphenol oxidase (PPO), peroxidase (POD), pectinase, cellulase, and protease were determined by corresponding colorimetric methods. Enzyme activities were expressed as the change in absorbance per minute per gram of sample, and all assays were performed in triplicate.

PPO activity was determined using catechol as the substrate according to the catechol colorimetric method [22]. Absorbance was recorded at 525 nm. One unit of PPO activity (U/g) was defined as the amount of enzyme that caused an increase of 0.005 in absorbance at 525 nm per minute per gram of tissue per milliliter of reaction mixture.

POD activity was measured using the guaiacol colorimetric method in the presence of H_2_O_2_ [23]. Absorbance was recorded at 470 nm. One unit of POD activity (U/g) was defined as the amount of enzyme that caused an increase of 0.005 in absorbance at 470 nm per minute per gram of tissue per milliliter of reaction mixture.

Pectinase and cellulase activities were determined using the 3,5-dinitrosalicylic acid (DNS) colorimetric method using pectin and carboxymethyl cellulose as substrates, respectively. One unit of pectinase activity (mg/h/g) was defined as the amount of enzyme that released 1 mg of galacturonic acid from pectin per gram of sample per hour under the assay conditions (50 °C, pH 3.5). One unit of cellulase activity (µg/min/g) was defined as the amount of enzyme that catalyzed the release of 1 µg glucose per minute per gram of sample.

Protease activity was determined using casein as the substrate using the phosphomolybdic acid colorimetric method [24]. One unit of protease activity (nmol/min/g) was defined as the amount of enzyme that hydrolyzed casein to release 1 nmol tyrosine per minute per milligram of sample at 30 °C.

### 2.5. Sample Extraction

Referring to previously reported methods [25], aroma components in tea samples were extracted using the stir bar sorptive extraction (SBSE) technique. Specifically, 600 mg of ground tea and 500 mg of sodium chloride were placed into a sample vial, followed by the addition of 10 mL of hot water at 80 °C for infusion. After allowing the mixture to equilibrate for 1 min, a PDMS stir bar (Gerstel, Mülheim, Germany; length 10 mm, thickness 0.5 mm, volume 24 μL) was immersed in the tea infusion for extraction using a magnetic stirrer (Thermo Fisher Scientific, Waltham, MA, USA, Advanced Multipoint 6/15) at 1250 rpm for 90 min. After extraction, the stir bar was rinsed with distilled water, gently wiped dry with lint-free tissue, and then transferred into a thermal desorption tube for subsequent analysis.

### 2.6. GC-MS Analysis

Referring to a previously established methodology [25], analysis was performed using an Agilent 6890A gas chromatography system coupled with a 5975C mass spectrometer (Agilent Technologies, Santa Clara, CA, USA). High-purity helium (purity > 99.99%) was used as the carrier gas at a constant flow rate of 1.6 mL/min in solvent vent mode. Separation was achieved using an HP-5MS capillary column (30 m × 250 μm × 0.25 μm, Agilent Technologies) with the following temperature program: initial temperature 50 °C (held for 2 min), ramped to 170 °C at 4 °C/min (held for 5 min), and then increased to 265 °C at 10 °C/min (held for 5 min). For mass spectrometric detection, electron impact (EI) ionization was applied with the following parameters: transfer line temperature, 280 °C; ion source temperature, 220 °C; ionization energy, 70 eV; and mass scan range, 30–600 AMU.

Volatile compounds were tentatively identified through multi-dimensional matching: mass spectra were compared with reference libraries (mainlib, replib, and NIST retention index database), while retention indices and retention times were verified against the published literature and an online database (https://webbook.nist.gov/chemistry/cas-ser.html, accessed on 17 October 2025). Retention indices were calculated under identical experimental conditions using a homologous series of n-alkanes (C3–C7, C8–C40). Quantitative analysis of volatile compounds was performed using an internal standard-based semi-quantitative approach. Ethyl decanoate was selected as the internal standard. Based on its density (0.871 g/mL), ethyl decanoate was dissolved in ethanol to prepare a stock solution at 1000 mg/L, which was further diluted to obtain a working solution of 100 mg/L. During sample analysis, 10 μL of the working solution was added to each 10 mL sample, resulting in a final internal standard concentration of 100 μg/L; the same amount of internal standard was added to all samples. Volatile compounds were quantified by calculating the ratio of their chromatographic peak areas to that of the internal standard, and the results were expressed in μg/L.

### 2.7. Data Processing and Statistical Analysis

All physicochemical component analyses were performed with three independent biological replicates. For each biological replicate, measurements were carried out with triplicate technical replicates. The results were expressed as the mean ± standard deviation (SD) of the three biological replicates (n = 3). The determination of volatile aroma compounds was conducted with four independent biological replicates. Principal component analysis (PCA), partial least squares-discriminant analysis (PLS-DA), hierarchical cluster analysis (HCA), and partial least squares analysis (PLS) were performed using Simca (version 14.1, Umetrics AB, Umeå, Sweden). Heatmap analysis was performed using TBtools (version v1.098).

## 3. Results and Discussion

### 3.1. Changes in Major Chemical Constituents

#### 3.1.1. Polyphenols

**Figure 1:**
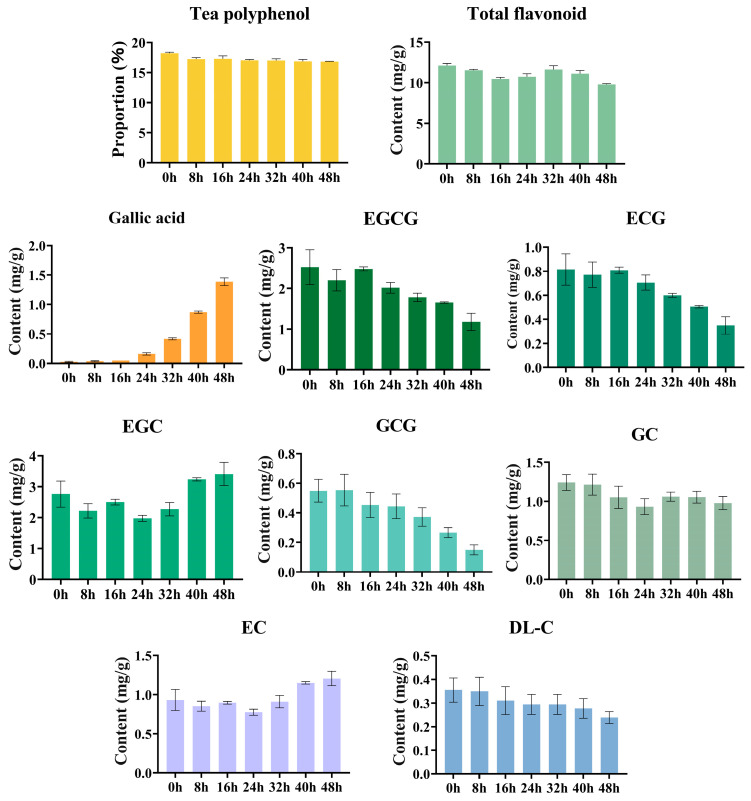
Dynamic changes in the content of polyphenolic components during pile fermentation of primary dark tea.

During the early stage of pile fermentation (0–16 h) (Table S2, Figure 1), both tea polyphenols and total flavonoids exhibited a slight decline. Ester-type catechins such as EGCG and ECG showed a continuous decreasing trend, whereas non-ester catechins (EGC and EC) first decreased and then increased. Meanwhile, gallic acid accumulated progressively, indicating the initial activation of ester bond hydrolysis. At this stage, endogenous esterases/tannases together with extracellular tannases secreted by microorganisms constitute the major catalytic system, driving the de-esterification of ester-type catechins (EGCG/ECG) to generate EGC, EC, and gallic acid, thereby providing precursor substrates for subsequent oxidation reactions [26]. During the mid-stage of pile fermentation (24–32 h), EGCG, ECG, and GCG continued to decrease, total flavonoids further declined, and gallic acid accumulated markedly; concurrently, the levels of EGC and EC increased substantially. These results suggested that rapidly proliferating exogenous microorganisms secrete high-activity tannase/esterase and other hydrolases during this phase, thereby intensifying the de-esterification of ester-type catechins [10,11]. During the late stage (32–48 h), EGCG, ECG, and GCG decreased to their lowest levels, while gallic acid accumulated sharply (up to 1.388 mg/g), and both EGC and EC became significantly higher than their initial concentrations. This pattern indicated that part of the monomeric phenolics has undergone oxidation or polymerization into high-molecular-weight pigments such as theaflavins and thearubigins [26,27]. Collectively, the quantitative evidence clearly demonstrated that the transformation of phenolic compounds during the pile fermentation of primary dark tea is dominated by ester bond hydrolysis (de-esterification), which was accompanied by secondary oxidation and polymerization processes. This conclusion is primarily drawn from the pronounced decrease in the content of esterified catechins (e.g., EGCG and ECG) alongside a significant increase in their hydrolytic products (e.g., EGC, EC, and gallic acid) throughout the fermentation process, as detailed in the quantitative analysis.

#### 3.1.2. Water Extract, Amino Acids, and Alkaloids

**Figure 2:**
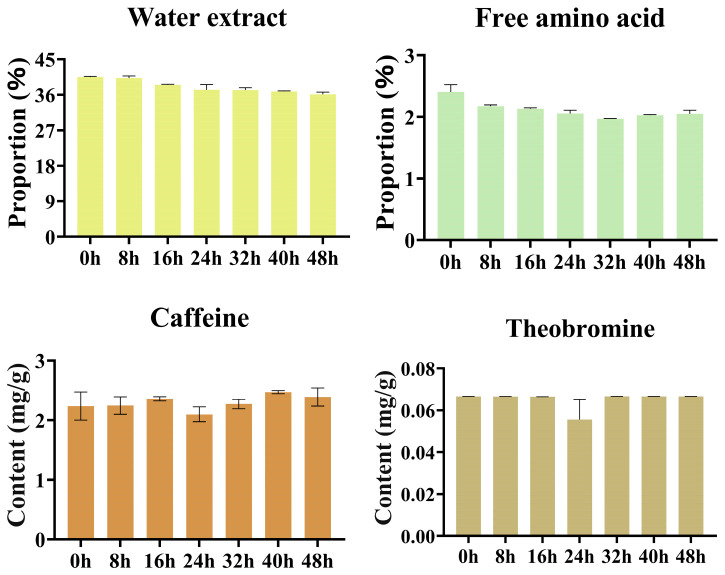
Changes in water extract, free amino acids, and alkaloids during primary dark tea pile fermentation.

During the pile fermentation process (Table S2, Figure 2), the contents of water extract and free amino acids decreased overall, whereas caffeine and theobromine remained relatively stable. In the early stage (0–16 h), microorganisms were in an adaptive growth phase, consuming soluble sugars and free amino acids as energy and nitrogen sources, resulting in slight reductions in both components. During the mid-stage (16–32 h), microbial metabolic activity intensified. The water extract content decreased from 38.49% to 37.19% and free amino acids dropped to their minimum level (1.97%), indicating the sustained depletion of carbon and nitrogen sources. However, the declining trend of water extract content slowed between 24 h and 32 h, likely due to the extensive degradation of cell wall polysaccharides (such as pectin and cellulose), which generated new soluble sugars and pectic fragments that partially compensated for microbial consumption [28,29,30]. Additionally, the reduction in free amino acids may also be attributed to their involvement in the PPO- and POD-mediated oxidation of catechins. Amino acids participate as quinone intermediates in the formation of colored polymers and flavor precursors, thereby contributing to color development and aroma precursor formation [2,27]. In the late stage, the system approached a steady state. Water extract and free amino acid contents stabilized at relatively low levels (decreasing by 10.9% and 14.9%, respectively, compared with 0 h). Nevertheless, free amino acids exhibited a slight increase (from 1.97% to 2.05%), which was presumably attributable to the continuous production of amino acids via protease-mediated hydrolysis. This “recovery” not only enhances the umami intensity of the tea infusion but also contributes to the development of a mellow and full-bodied taste in the later stages of fermentation [2,31]. The contents of caffeine and theobromine remained stable throughout the process, indicating that purine alkaloids are chemically robust and do not serve as major metabolic substrates during pile fermentation [32].

#### 3.1.3. Proteins and Polysaccharides

**Figure 3:**
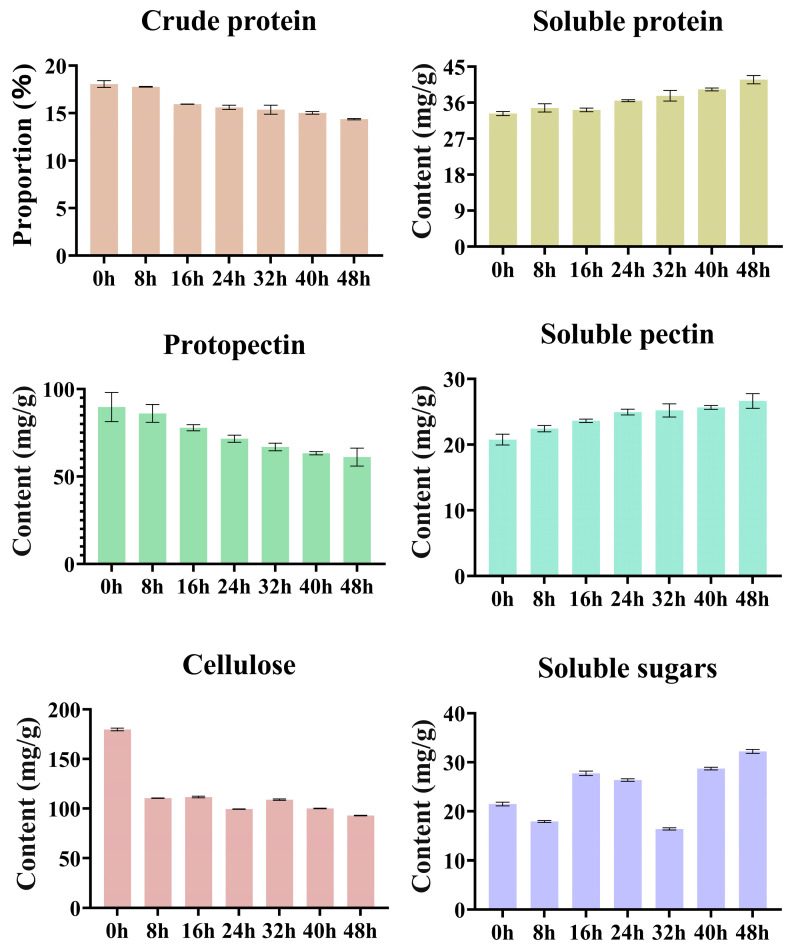
Analysis of proteins and polysaccharides during pile fermentation of primary dark tea.

During the pile fermentation process (Table S2, Figure 3), structural components such as crude protein, protopectin, and cellulose exhibited pronounced changes that closely aligned with the activities of their corresponding enzymes, while flavor components including soluble protein, soluble pectin, and soluble sugars exhibited complex accumulation dynamics. In the early stage, the crude protein content decreased (from 18.06 to 15.95%), accompanied by a slight increase in soluble protein that indicated that proteases had initiated the hydrolysis of insoluble proteins into small molecular peptides and amino acids. Protopectin showed a marked reduction (from 89.72 to 77.87 mg/g), whereas soluble pectin increased concurrently (from 20.76 to 23.63 mg/g). This clear inverse relationship demonstrated the activation of pectinase and the initial loosening of cell wall structures. Meanwhile, cellulose content declined sharply (from 179.74 to 110.48 mg/g), and soluble sugars reached a consumption trough (from 21.49 to 17.92 mg/g).

During the mid-stage, crude protein continued to decline while soluble protein increased steadily, indicating a sustained enhancement of protease activity; this trend also corresponded to the early-stage depletion of free amino acids. The degradation rate of protopectin accelerated (from 77.87 to 66.89 mg/g), accompanied by an increase in soluble pectin to 25.20 mg/g. At the same time, cellulose content further decreased to 108.91 mg/g, whereas soluble sugars exhibited a pronounced fluctuation-driven rise (from 17.92 to 27.75 mg/g, followed by a slight decline). This pattern suggested that the rate of sugar release through cellulase-mediated hydrolysis begin to exceed the microbial respiratory consumption rate, leading to an intense dynamic equilibrium between sugar “generation” and “utilization”. The concurrent degradation of pectin and cellulose constitutes a major biochemical basis between the continuous softening of leaf tissues and enhancement of tea infusion viscosity [28,33,34]. During this period, soluble sugars experienced a second minor decline at 32 h (16.40 mg/g), which was likely attributable to the renewed rapid microbial uptake of the newly released carbohydrate substrates.

During the late stage of pile fermentation, crude protein continued to decline (from 15.36% to 14.37%), whereas soluble protein accumulated markedly (from 37.72 to 41.74 mg/g), indicating the persistence of protease-dominated hydrolysis. Protopectin further decreased to 61.09 mg/g, while soluble pectin increased to 26.65 mg/g, though with a reduced rate of increase, suggesting that pectin degradation was approaching its endpoint and that the generation–consumption processes had reached a dynamic equilibrium. Cellulose content steadily decreased to 92.88 mg/g, reflecting sustained enzymatic hydrolysis. Soluble sugars ultimately accumulated to 32.22 mg/g, demonstrating that the late-stage hydrolysis rate exceeded the microbial consumption rate, resulting in a characteristic “initial depletion followed by accumulation” pattern. During the entire pile fermentation process, crude protein and protopectin decreased by approximately 20.4% and 31.9%, respectively, whereas soluble protein and soluble pectin increased by 25.5% and 28.3%, highlighting a pronounced trend of enzymatic hydrolysis and solubilization. The decrease in cellulose was even more substantial (approximately 48.3%), and soluble sugars accumulated by about 50% by the end of fermentation. The coordinated degradation of proteins, pectins, and cellulose not only supplied abundant nitrogen- and carbon-containing substrates for microbial metabolism but also generated a large quantity of soluble constituents, thereby establishing the essential material basis for its mellow, smooth mouthfeel characteristic of primary dark tea [33].

### 3.2. Enzyme Activity Analysis

**Figure 4:**
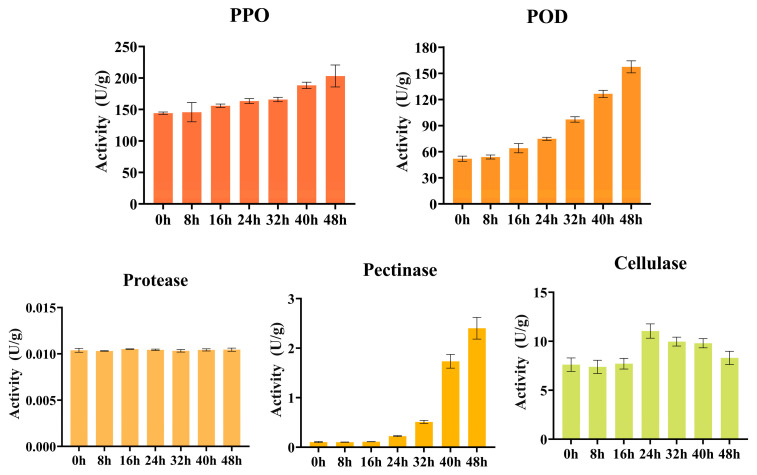
Analysis of enzyme activity analysis during pile fermentation of primary dark tea.

During the pile fermentation process (Table S2, Figure 4), the activities of various enzymes exhibited significant stage-specific variations. In the early phase (0–16 h), both PPO and POD activities increased steadily (PPO: from 144.17 to 155.97 U/g; POD: from 51.97 to 64.03 U/g), indicating the initiation of phenolic oxidation. Concurrently, pectinase and cellulase activities, though low, began to emerge, demonstrating the initial deconstruction of cell wall structures—consistent with the observed decrease in protopectin and increase in soluble pectin during this phase. Notably, cellulase activity was already maintained at a baseline level of 7.61 U/g during this phase, directly contributing to the sharp reduction in cellulose content (from 179.74 to 111.64 mg/g). Correspondingly, soluble sugars exhibited a transient decline at 8 h followed by a rebound at 16 h (from 21.49 to 27.75 mg/g).

The mid-stage (16–32 h) represented the core phase of enzymatic reactions and structural reconfiguration. During this period, PPO and POD activities increased markedly, corresponding to the peak of phenolic oxidation and polymerization—an essential process driving the transformation of tea liquor color from green to yellowish-brown and the shift in taste from astringent to mellow [26,27,32]. This temporal pattern aligned closely with the rapid depletion of phenolic compounds and the concurrent accumulation of gallic acid. Pectinase activity increased substantially (from 0.112 to 0.511 U/g), consistent with the pronounced reduction in protopectin and the rise in soluble pectin observed earlier. Cellulase activity reached its maximum at 24 h (11.04 U/g). During this period, the synergistic actions of cellulase and pectinase induced extensive cell wall degradation, which not only explains the further decline in cellulose content (from 111.64 to 99.45 mg/g) but also lead to a significant rebound and accumulation of soluble sugars (from 17.92 to 27.75 mg/g). This process provides essential substrates for enhancing sweetness and facilitating Maillard reactions [33,34].

During the late stage, the activities of PPO and POD increased to their highest levels (PPO: 203.23 U/g; POD: 157.50 U/g). At this stage, the oxidation and polymerization of phenolic compounds entered an intensified phase, promoting the formation of pigments such as theaflavins and thearubigins [26,35]. Pectinase activity continued to rise (up to 2.40 U/g), whereas cellulase activity showed a slight decline; however, cellulose content continued to decrease, suggesting substrate depletion or feedback inhibition. Soluble sugars accumulated significantly to 32.22 mg/g in the late stage, demonstrating a characteristic pattern of “mid-phase consumption followed by late-phase accumulation due to dominant hydrolysis”. Pectinase activity increased sharply after 24 h and peaked at 48 h, while cellulase reached a relatively high level at 24 h. The activity peaks of both enzymes coincided with the continuous decline in protopectin and the partial replenishment of soluble pectin and water-extractable substances. Protease activity remained consistently low throughout the process, indicating that protein transformation relied more on microbial metabolism and peptidase activity rather than direct proteolysis. These findings are consistent with the observations of Li et al. [36], who similarly reported enzyme-related mechanisms underlying quality formation during the pile fermentation of dark tea.

### 3.3. Identification and Quantification of Volatile Compounds in Primary Dark Tea During Pile Fermentation

**Figure 5:**
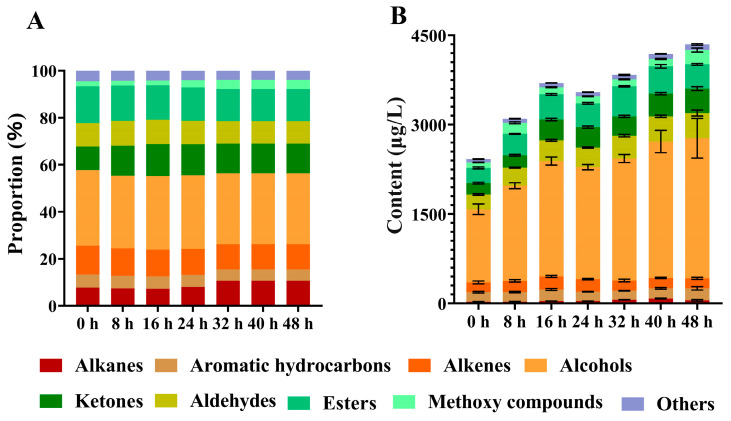
Dynamic changes in the volatiles relative proportion (**A**) and content (**B**) during the pile fermentation of primary dark tea.

To investigate the dynamic changes in volatile compounds during the pile fermentation of dark tea, the tea samples collected at different fermentation stages were analyzed using GC-MS. A total of 245 volatile compounds were preliminarily detected after peak alignment. Considering that the automatic MS analysis by the software may contain errors, volatile compounds were further verified by RI and standards. RI comparison and validation using standards resulted in 103 dependable volatile compounds. In total, 103 volatiles were identified (Table S3), comprising 11 alkanes, 5 aromatic hydrocarbons, 11 alkenes, 31 alcohols, 13 aldehydes, 10 ketones, 14 esters, 4 methoxy compounds, and 4 other compounds (Figure 5A).

During the pile fermentation process (Figure 5B), the total content of aroma compounds exhibited a continuous and pronounced increase. This observation confirmed that pile fermentation is a critical stage for the formation and enrichment of aroma constituents in dark raw tea. Among the nine categories of volatile compounds identified, alcohols constituted the highest proportion, accounting for 50.7–54.1% of the total volatiles. Linalool was the most abundant alcohol (490.56–618.77 μg/L), representing 20.2–26.3% of the alcohol fraction, followed by phytol (224.18–618.77 μg/L) and nerolidol (140.39–196.08 μg/L). Esters comprised 10.6–13.1% of the total volatiles, with methyl salicylate (87.76–306.65 μg/L) being the predominant compound in this class, accounting for 34.2–75.2% of the ester fraction. Aldehydes represented 6.4–9.0% of the total, while ketones increased from an initial 7.9% to 9.4% in the later stage. In contrast, olefins, aromatic hydrocarbons, alkanes, and methoxy-substituted compounds collectively accounted for less than 20% of the total aroma profile. Throughout the fermentation process, methoxy-substituted compounds, ketones, alcohols, alkanes, esters, aldehydes, and other compound categories increased by 161.7%, 114.6%, 91.2%, 97.9%, 58.8%, 71.5%, 69.5%, 2.2%, and 25.4%, respectively.

**Figure 6:**
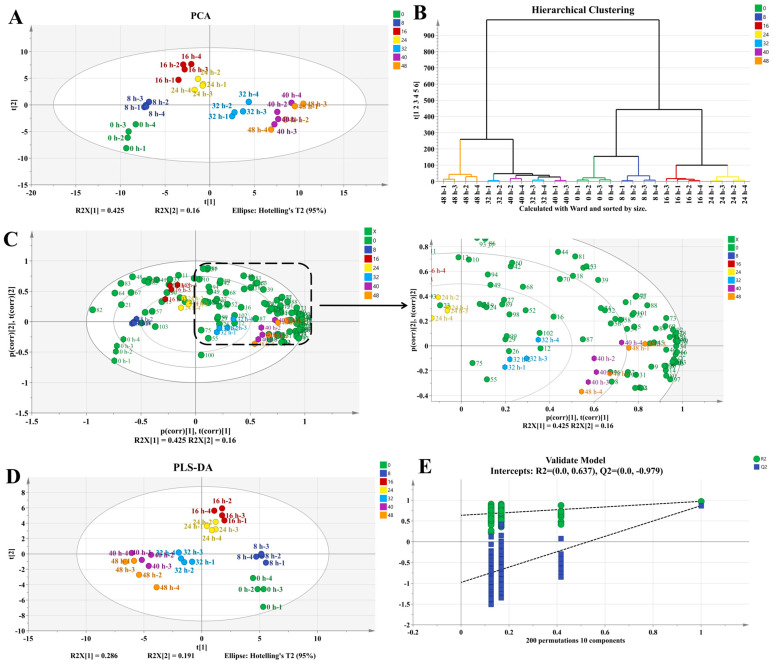
Multivariate analysis of volatile compounds during pile fermentation: (**A**): PCA of primary dark tea samples. (**B**): Hierarchical clustering analysis (HCA). (**C**): PLS of the relationship between pile fermentation duration and active aroma compounds in primary dark tea. (**D**): Score plot of PLS-DA. (**E**): Permutation test of the PLS-DA model (200 permutations).

To further elucidate the dynamic patterns of aroma compounds during the pile fermentation process, PCA and HCA analyses were performed (Figure 6A,B). Both PCA and HCA revealed clear distinctions among different fermentation stages and clarified the relationships among samples at various fermentation levels. The markedly different scatter distributions between the 0 h and 48 h samples indicated substantial shifts in volatile profiles throughout the process. Based on these multivariate results, the pile fermentation process can be divided into three distinct stages: an early stage (0–16 h), a middle stage (24–32 h), and a late stage (32–48 h), which is consistent with the trends observed in the physicochemical analyses. As shown in Figure 6C, 3-methyltridecane, heptadecane, (3*E*)-4,8-dimethyl-1,3,7-nonatriene, α-farnesene, 1-butanol, 1-octen-3-ol, benzaldehyde, cis-3-hexenoate, 1,2,3-trimethoxybenzene, and linalool contributed significantly to the differentiation among fermentation stages. Moreover, a total of 59 differential aromatic compounds, including 5 alkanes, 4 aromatic hydrocarbons, 8 alkenes, 18 alcohols, 8 aldehydes, 5 ketones, 6 esters, 3 methoxy compounds, and 2 other compounds (Table S4), were identified using the PLS-DA model (VIP > 1 and *p* < 0.05) (Figure 6D). To further assess the risk of overfitting, a permutation test with 200 iterations was conducted. The results (Figure 6E) show that the regression lines of the permutation test start at (R2 = 0.973, Q2 = 0.869) when the correlation is 1, with *Y*-axis intercepts at (R2 = 0.637, Q2 = −0.970). Both regression lines exhibit a significantly negative slope. This pattern aligns with the criterion for model robustness, indicating that the separation results of the original model are not due to overfitting.

### 3.4. Multivariate Analysis of Volatile Compounds

#### 3.4.1. Alcohols

**Figure 7:**
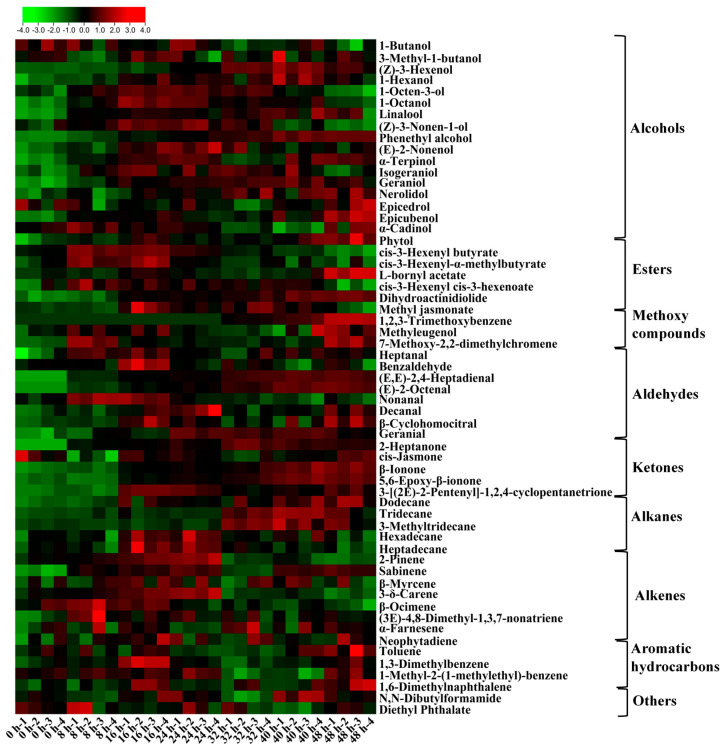
Heatmap analysis of critical metabolites (VIP > 1) during the pile fermentation of primary dark tea. Color scale indicates Z-score normalized abundance (green: low; red: high).

Alcohols exhibited a continuously increasing trend throughout the pile fermentation process (Figure 7). Linalool, geraniol, nerolidol, and phytol were the predominant constituents, accounting for the major proportion of total alcohols, thereby highlighting their central roles in the aroma formation of primary dark tea. Linalool, a key contributor to the floral notes of dark tea [10], increased from 490.56 μg/L to 706.91 μg/L and remained at a high level after peaking at 40 h. This pattern suggested the substantial release of its precursors through oxidative hydrolysis during the peak activity phase of PPO [32,37]. Geraniol and nerolidol also accumulated steadily, reaching 180.31 μg/L and 196.08 μg/L at 48 h, respectively, imparting pronounced floral, fruity, and sweet attributes to the tea [38,39]. Meanwhile, the phytol content increased from 224.18 μg/L to 618.77 μg/L, indicating enhanced lipid degradation and chlorophyll side chain cleavage, which corresponded to the high-activity phase of pectinase and cellulase (32–48 h). Among medium- and low-molecular-weight alcohols, phenethyl alcohol showed a remarkable accumulation and reached 181.68 μg/L at 48 h, closely associated with the degradation of aromatic amino acid precursors under protease activity [38,40,41]. In contrast, short-chain alcohols (e.g., 1-butanol, 1-hexanol) exhibited slight fluctuations during the early stage, whereas (*Z*)-3-hexenol increased markedly during 32–40 h (up to 78.05 μg/L). Overall, the pronounced increase in alcohols—driven by lipid degradation, chlorophyll cleavage, and proteolysis—constituted a key biochemical basis for the transformation of aroma from “green and grassy” to “mellow and floral” during pile fermentation [39,41].

#### 3.4.2. Esters and Methoxy Compounds

During the pile fermentation process, ester compounds exhibited a dynamic change by first rising and then falling. In the early stage (0–16 h), C6 esters represented by cis-3-hexenyl butyrate (from 12.40 to 19.72 μg/L) and cis-3-hexenyl cis-3-hexenoate (from 5.17 to 6.84 μg/L), increased significantly, contributing fresh green and fruity aroma characteristics to primary dark tea [10,36,41]. Subsequently, their content gradually decreased, indicating the conversion of C6 esters into oxides and aromatic ketones, reflecting a trend of “greenness dissipation” [38,39,41]. Meanwhile, L-bornyl acetate showed a notable increase in the later stage, while dihydroactinidiolide, which imparts an aged aroma, demonstrated a continuous accumulation trend, suggesting enhanced lipid oxidation and carotenoid degradation [36].

Aromatic methoxy compounds accumulated significantly during the middle and late stages of pile fermentation. 1,2,3-Trimethoxybenzene was nearly undetectable from 0 to 24 h but accumulated in the middle and later stages, reaching 35.64 μg/L at 48 h. Methyleugenol overall remained stable with minor fluctuations (from 1.755 μg/L to 2.438 μg/L), indicating its high stability within the pile fermentation system. 7-Methoxy-2,2-dimethylchromene reached its first peak of 185.219 μg/L at 8 h, experienced a plateau period, and eventually increased to 193.200 μg/L by 48 h. The accumulation of these compounds is associated with the oxidative polymerization of phenolics and microbial methoxylation metabolism. This corresponds to enhanced activity in phenolic transformation and oxidative condensation reactions during the middle and late fermentation stages, being the key source of “aged aroma” and “medicinal aroma” substances [1,42].

#### 3.4.3. Aldehydes and Ketones

Aldehyde compounds typically impart floral and fruity aroma characteristics [43,44]. During the pile fermentation process, the contents of aldehydes such as nonanal, benzaldehyde, heptanal, decanal, geranial, and (*E*,*E*)-2,4-heptadienal were relatively high, a finding consistent with previous literature reports [45]. The dynamics of these aldehydes during fermentation followed three distinct patterns. The first is the type with a peak in the early stage followed by decline. This is exemplified by nonanal and benzaldehyde, which reached transient peaks at 8–16 h (nonanal: 123.677 μg/L at 8 h; benzaldehyde: 108.891 μg/L at 16 h) before decreasing and stabilizing near their initial levels. The second is the type with a peak in the middle stage, represented by heptanal, decanal, and geranial, which generally peaked during 16–24 h (e.g., geranial reached 21.421 μg/L at 24 h) and subsequently declined partially but remained significantly higher than their starting concentrations. The third is the type of continuous accumulation/second peak. Characteristic of conjugated unsaturated aldehydes, such as (*E*,*E*)-2,4-heptadienal and (*E*)-2-octenal, these compounds accumulated rapidly in the middle to late stages (32–40 h) and reached high levels by 40–48 h. These three stages correspond to the release of initial fruity and nutty scents, the formation of lipid oxidation-dominated citrus–herbaceous scents, and the final establishment of a green-aged aroma foundation [13,45,46].

Among ketone compounds, β-ionone and 5,6-epoxy-β-ionone increased continuously (from 33.93 to 93.20 μg/L and from 24.58 to 54.60 μg/L, respectively). This upward trend is closely associated with carotenoid cleavage [10], contributing to the typical sweet and mellow flavors of dark tea such as violet and woody scents [10]. 3-[(2*E*)-2-Pentenyl]-1,2,4-cyclopentanetrione reached its peak at 16 h (157.83 μg/L), being a mid-stage conversion product. 2-Heptanone, initially undetectable (0 h), gradually accumulated to 2.59 μg/L by the end of fermentation (48 h). Overall, the formation of aldehydes and ketones reflected a progressive pathway—from short-chain lipid oxidation products to aromatic conjugated oxides, and further to carotenoid degradation compounds [47]—synchronizing with the PPO/POD activity curves and phenolic oxidation polymerization.

#### 3.4.4. Alkanes and Alkenes

During the pile fermentation process, most alkane compounds exhibit an overall increasing trend, with the notable exceptions of hexadecane and heptadecane. Dodecane, for instance, increased continuously and reached its maximum concentration (8.50 μg/L) at 48 h. Both tridecane and 3-methyltridecane peaked at 40 h, a period coinciding with enhanced activities of pectinase and cellulase, suggesting that alkane release was synchronized with cell wall degradation. In contrast, hexadecane and heptadecane showed relatively stable profiles, reflecting their higher chemical stability and lower reactivity.

During the pile fermentation process, the terpene compounds exhibited a significant staged evolution pattern. Representative monoterpenes, namely 2-pinene and 3-δ-carene, reached their peak concentrations (58.69 μg/L and 39.37 μg/L, respectively) at 24 h, followed by a subsequent decline. In contrast, β-myrcene (increasing from 11.81 μg/L to 12.98 μg/L) and sabinene (from 1.41 μg/L to 4.28 μg/L) showed a fluctuating yet overall upward trend. Meanwhile, the sesquiterpene α-farnesene (from 12.21 μg/L to 14.11 μg/L) and the diterpene neophytadiene (from 28.80 μg/L to 37.56 μg/L) increased in content in the later stage of fermentation. This rebound indicated a reactivation of both lipid degradation and isoprenoid synthesis pathways, which—together with the disruption of lipid membrane structures and amino acid degradation—collectively contribute to the development of the characteristic “aged aroma” [47,48]. The transformation of these terpenoid species was highly synchronized with the enhanced activity of PPO and POD, reflecting the dominant role of the oxidase system in driving aroma maturation during the later phases of fermentation [36].

#### 3.4.5. Aromatic Hydrocarbons

During the pile fermentation, toluene (from 98.06 μg/L to 119.41 μg/L) and 1,3-dimethylbenzene (from 44.94 μg/L to 53.51 μg/L) exhibited an overall fluctuating yet increasing trend, while 1-methyl-2-(1-methylethyl)-benzene (from 12.07 μg/L to 12.75 μg/L) remained relatively stable throughout the process. 1,6-dimethylnaphthalene (from 4.85 μg/L to 7.82 μg/L) showed a characteristic late-stage accumulation pattern, reaching its peak at 48 h. These compounds are largely formed through the oxidative condensation of phenolic compounds or the cyclization of aliphatic carbon skeletons, consistent with the enhanced oxidative polymerization reactions occurring in the later stages of pile fermentation [47].

## 4. Discussion

The pile fermentation of primary dark tea is a complex biochemical reaction system driven primarily by microbial activity, enzymatic action, and synergistic thermo-humid effects. This study confirmed that the transformation of phenolic compounds during this process follow a pattern characterized by “predominant ester bond hydrolysis, supplemented by oxidative polymerization”. The degradation rates of EGCG and ECG, along with the accumulation rate of gallic acid, increased markedly after 24 h. This timeframe corresponds with the peak activity periods of PPO and POD, indicating that enzymatic oxidation plays a more critical role than simple hydrolysis in the middle to late fermentation stages. This finding is consistent with the quality transformation patterns reported by Li et al. [39] for Pu-erh tea fermentation. However, primary dark tea exhibited a faster ester bond cleavage rate and higher gallic acid accumulation efficiency, suggesting that its microbial community and process conditions may have a stronger activating effect on the enzymatic system. It thus inferred that the concurrent hydrolysis and oxidation of catechins during the pile fermentation of primary dark tea not only form the chemical basis for the color transition, but also establish the material foundation for the subsequent formation of aroma precursors and flavor-active compounds [8,10,49]. Notably, the observed decrease in total phenols and total flavonoids was substantially less pronounced than the degradation of esterified catechins, which suggests that besides catechin transformation, other processes are occurring, such as the hydrolysis of other phenolic substances like flavonoid glycosides, and the formation of oxidative polymerization products like theaflavins and thearubigins [26]. This observation aligns with studies by Kelebek et al. [50] on the conversion of phenolic compounds into tea pigments during tea fermentation.

This study observed the synchronous degradation of crude protein and protopectin during the pile fermentation process, accompanied by a corresponding increase in soluble protein and soluble pectin. This clear “see-saw” relationship was highly synchronized with a sharp surge in pectinase and cellulase activities, indicating that the extensive deconstruction of cell wall structures is one of the core events during pile fermentation. This process corresponds with the “proteolysis–pectin degradation–aroma formation” model observed by Li et al. [49] in dark tea fermentation systems. Compared with the study by Li et al. [51] the present study found that protease activity remained at a relatively low level throughout the process, suggesting that protein transformation may rely more on microbial extracellular peptidase systems rather than direct plant-derived proteases.

This study further elucidates the driving mechanism behind aroma quality formation. The conversion of alcohols and esters was closely synchronized with the peak activity of pectinase and the cell wall degradation process. Esters exhibited an initial increase followed by a decrease, reflecting the chemical transition from “greenness reduction” to “aging development”. Aldehydes and ketones demonstrated three distinct patterns, early-stage peaking, mid-stage peaking, and late-stage accumulation, revealing the phased characteristics of lipid oxidation. The enrichment of late-stage unsaturated aldehydes and carotenoid-derived ketones (e.g., β-ionone) coincided with the high-activity period of PPO/POD and the emergence of an “aged aroma”, indicating that the synergy between phenolic oxidation and lipid degradation is crucial for the formation of dark tea’s characteristic aged scent. This research clarifies the division and coordination of different enzyme systems in aroma development: pectinase and cellulase synergistically promote cell wall disintegration and substrate release, while PPO/POD drive oxidation reactions that generate various floral, fruity, and aged aroma compounds. The temporal consistency between enzyme activity dynamics and chemical compositional changes demonstrates that the pile fermentation of primary dark tea is a multi-enzyme coordinated metabolic system centered on “oxidation–hydrolysis–reconstruction”. Consistent with multi-omics studies on ripened Pu-erh tea and Heizhuan tea [7,10,38,39], the enzyme activity peaks in primary dark tea occurred earlier, suggesting higher efficiency in process control (temperature/humidity) and microbial ecology regulation.

It should be noted that the associations between volatile compounds identified in this study and specific sensory notes are primarily inferred from the existing literature. While this provides valuable clues for understanding potential aroma formation, the direct sensory evaluation and calculation of odor activity values (OAVs) for the tea samples studied here are required in future work to conclusively establish their contributions to the perceived aroma.

## 5. Conclusions

This study systematically elucidates the quality formation mechanisms during the pile fermentation of primary dark tea. The results demonstrated significant de-esterification and re-oxidative polymerization of phenolic compounds, with esterified catechins decreasing substantially while non-esterified catechins and gallic acid increased markedly. The enzymatic hydrolysis of proteins and polysaccharides led to notable reductions in crude protein, protopectin, and cellulose, alongside significant increases in soluble protein, soluble pectin, and soluble sugars. PPO and POD dominated the oxidative polymerization of phenols, while pectinase and cellulase accelerated cell wall disintegration and the release of volatile precursors. Proteases supplied substrates such as aromatic amino acids for aroma formation. These enzymatic activities exhibited temporal patterns highly consistent with the transformation of chemical components. This process altered the tea’s texture and supplied substrates for subsequent aroma formation. A total of 103 volatile compounds were qualitatively and quantitatively analyzed by GC-MS, covering nine major categories, with alcohols, esters, aldehydes, and ketones being the main contributing components. The total aroma content increased significantly during fermentation. Floral and fruity components such as linalool and geraniol accumulated continuously, while esters showed an initial increase followed by a decrease, with grassy esters gradually replaced by aged aroma substances. The degradation products of carotenoids represented by β-ionone significantly accumulated in the later stage. Collectively, these findings clarify that the quality transition from “green and astringent” to “mellow and aged” is underpinned by an integrated biochemical cascade: initial de-esterification and hydrolysis lay the material foundation, while coordinated enzymatic actions drive the conversion, culminating in a defined volatile profile. This mechanistic insight provides a theoretical foundation for the scientific regulation of the pile fermentation process.

These insights establish a clear foundation for future work: theoretical research should focus on validating the specific roles of key enzymes and tracing aroma precursor pathways, while the practical application lies in regulating fermentation by monitoring the identified chemical and enzymatic markers to directionally enhance the desired aroma compounds and improve product quality.

## Data Availability

The original contributions presented in this study are included in the article/Supplementary Material. Further inquiries can be directed to the corresponding authors.

## Supplementary Materials

The following supporting information can be downloaded at: https://www.mdpi.com/article/10.3390/foods15020212/s1, File S1: Detailed protocols for the determination of water extract, polyphenols, amino acids, total flavonoid, crude protein, soluble protein, protopectin, soluble pectin, cellulose, and soluble sugars; Figure S1: Standard and Sample Chromatograms: A: Standards. B: Samples (0–48 h); Table S1: The calibration curves for each target compound; Table S2: Dynamic changes of key chemical components during pile fermentation of dark raw tea; Table S3: Information on 103 Aroma Compounds (μg/L); Table S4: List of identification information of the key differential substances (μg/L).
