# Supplementary material for: Multidimensional Analysis Reveals the Flavor Quality Formation Mechanism During the Primary Pile Fermentation of Dark Tea
Source: Foods. 2026 Jan 7;15(2):212. doi: 10.3390/foods15020212 (PMC12840366; doi:10.3390/foods15020212)
Supplement: Supplementary file 1 [file foods-15-00212-s001.zip › foods-4043439-supplementary.pdf]

Multidimensional Analysis Reveals the Flavor Quality Formation Mechanism during the Primary Pile Fermentation of Dark Tea

Dunchao Wu [1], Yufei He [2,3], Juanshu Wen [1], Hongfa Zheng [2,3], Xi Zhao [2,3], Penghui Yu [2,3], Ni Zhong [2,3], Li Niu [1,3,4], Shi Li [1,3,4], Yong Lin [1,3,4], Hao Huang [2,3,*], and Zhonghua Liu [1,3,4,*]

[1] Key Laboratory of Tea Science of Ministry of Education, Hunan Agricultural University, Changsha 410128, China; Dunchao_Wu@163.com (D.W.)
[2] Tea Research Institute, Hunan Academy of Agricultural Sciences, Changsha 410125, China
[3] Yuelushan Laboratory, Changsha, 410128, China
[4] National Research Center of Engineering Technology for Utilization of Functional Ingredients from Botanicals, Hunan Agricultural University, Changsha 410128, China
* Correspondence: haohuang_08@163.com (H.H.); larkin-liu@163.com (Z.L.)

Supplementary File S1. Detailed protocols for the determination of water extract, polyphenols, amino acids, total flavonoid, crude protein, soluble protein, protopectin, soluble pectin, cellulose, and soluble sugars.

(1) Determination of water extract (Based on GB/T 8305-2013)

Water extracts were determined using the boiling water reflux–gravimetric method. The principle of this method is that all water-soluble components in tea are fully extracted by boiling water, followed by filtration and drying of the residue. The content of water extracts is calculated based on the mass difference. Briefly, 2.0 g of the accurately weighed sample was extracted with 300 mL of boiling water in a 100 °C water bath for 45 min. After filtration, the tea residue was dried at 120 °C to constant weight, and the water extract content was calculated.

(2) Determination of polyphenols (Based on GB/T 8313-2018)

Polyphenols were determined using the Folin–Ciocalteu colorimetric method. The principle is that polyphenolic compounds can reduce the Folin–Ciocalteu reagent under alkaline conditions to form a blue complex, which exhibits a maximum absorbance at 765 nm. The absorbance is proportional to the polyphenol content. Briefly, samples were extracted with 70% methanol, and the extract was reacted with the Folin–Ciocalteu reagent and 7.5% sodium carbonate solution for 6 min (extract : Folin–Ciocalteu reagent : sodium carbonate = 1 : 5 : 4). After standing at room temperature for 60 min, the absorbance was measured at 765 nm, and quantification was performed using gallic acid as the standard.

(3) Determination of amino acids ((Based on GB/T 8314-2013)

Amino acids were determined using the ninhydrin colorimetric method. The principle of this method is that α-amino acids react with ninhydrin under pH 8.0 conditions upon heating to form a purple complex with a characteristic absorption at 570 nm. the aqueous sample extract was mixed with pH 8.0 phosphate buffer and 2% ninhydrin solution (sample:buffer:ninhydrin = 2:1:1) and heated in a boiling water bath for 15 minutes.. After cooling, the absorbance was measured at 570 nm, and quantification was carried out using theanine as the standard.

(4) Determination of Total Flavonoid

The total flavonoid content was determined using the aluminum chloride colorimetric method with rutin as the standard. Briefly, 1.0 g of ground tea sample was extracted with 20 mL of 70% methanol in a water bath at 70°C for 1 hour. After cooling and volumetric adjustment, 1 mL of the extract was mixed with 1 mL of 2.0% $AlCl_3$ solution. The mixture was allowed to react at room temperature in the dark for

10 minutes, and the absorbance was measured at 421 nm for quantification.

(5) Determination of crude protein (BCA method)

Approximately 0.1 g of tea sample was homogenized on ice with PBS extraction buffer (1:5–10, w/v) containing 1% SDS and 2% PVP. The homogenate was centrifuged at 10,000 rpm for 10 min at 4 °C, and the supernatant was mixed with 4–5 volumes of pre-cooled acetone and incubated at −20 °C overnight for protein precipitation. After centrifugation at 12,000 rpm for 15 min (4 °C), the protein pellet was redissolved in PBS containing 0.1% SDS. For determination, 4 μL of protein solution was mixed with 200 μL of BCA working reagent in a 96-well plate and incubated at 60 °C for 30 min. Absorbance was measured at 562 nm.

(6) Determination of soluble protein

Soluble protein content was determined using the BCA method with a commercial assay kit. Approximately 0.1 g of tea sample was homogenized in phosphate-buffered saline (PBS) at a ratio of 1:5-10, followed by centrifugation at 10,000 rpm for 10 min at 4 °C. Aliquots of the supernatant (4 μL) were reacted with 200 μL of BCA working reagent at 60 °C for 30 min. Absorbance was recorded at 562 nm, and protein content was quantified using the protein standard (0.5 mg/mL) supplied with the kit.

(7) Determination of protopectin

Protopectin content was determined using the carbazole colorimetric method. Approximately 0.05 g of tea sample was extracted with 1 mL extraction solution I at 90 °C for 30 min. After centrifugation and removal of the supernatant, the extraction was repeated once. The residue was then hydrolyzed with 1 mL extraction solution II at 90 °C for 1 h. The supernatant (100 μL) was reacted with carbazole reagent and sulfuric acid, and absorbance was measured at 530 nm. Galacturonic acid provided in the assay kit was used as the standard for quantification.

(8) Determination of soluble pectin

Soluble pectin content was measured using the carbazole colorimetric method. Approximately 0.1 g of tea sample was homogenized with extraction solution I and heated at 95 °C for 20 min. After centrifugation, the residue was washed alternately with extraction solution I and acetone, followed by extraction with solution II for 15 h. The extract was reacted with carbazole reagent and sulfuric acid, and absorbance was recorded at 530 nm. Results were calculated using galacturonic acid as the standard supplied with the kit.

(9) Determination of cellulose

Cellulose content was determined using the anthrone colorimetric method. Approximately 0.3 g of sample was extracted with 80% ethanol at 95 °C for 20 min to remove soluble components. The residue was washed with 80% ethanol and acetone, treated with reagent I for 15 h, and then hydrolyzed with concentrated sulfuric acid in an ice-water bath. After centrifugation and 20-fold dilution, absorbance was measured at 620 nm following reaction with anthrone reagent. Cellulose content was quantified using galacturonic acid supplied with the assay kit as the standard.

(10) Determination of soluble sugars

Soluble sugar content was determined by the anthrone colorimetric method. Approximately 0.1-0.2 g of sample was extracted with distilled water in a boiling water bath for 10 min. After centrifugation and dilution, the extract was reacted with anthrone–sulfuric acid reagent, heated at 95 °C for 10 min, and absorbance was measured at 620 nm. Soluble sugar content was calculated using glucose provided with the assay kit as the standard.

A

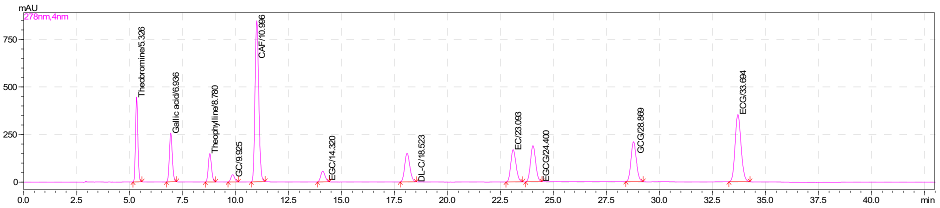

B-0 h

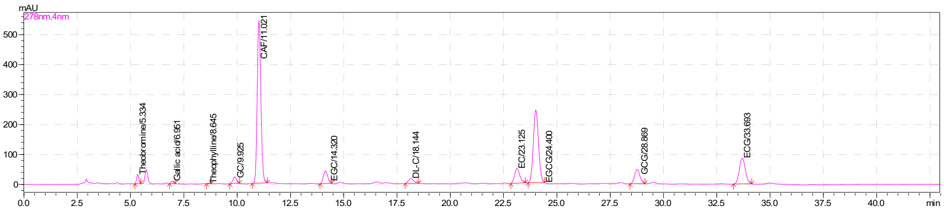

B-8 h

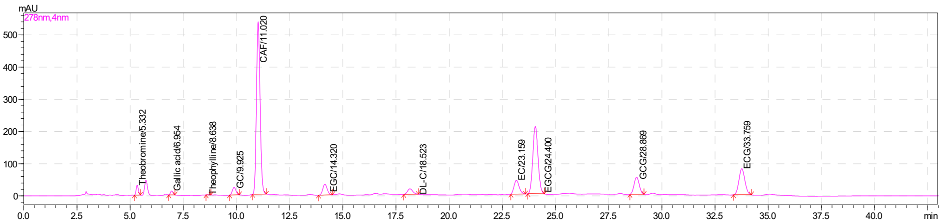

B-16 h

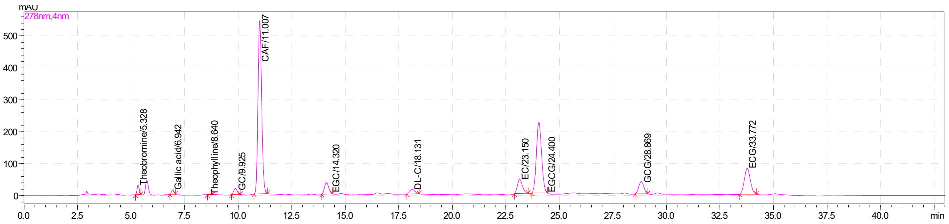

B-24 h

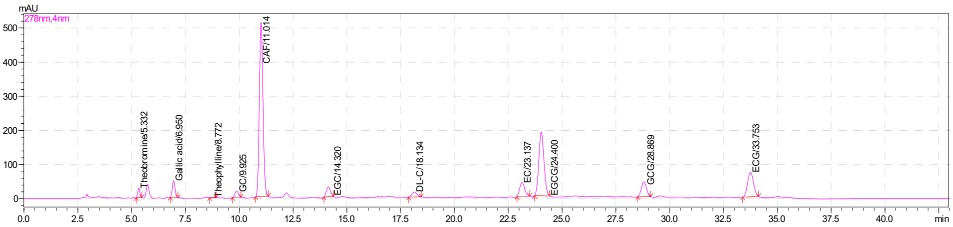

B-32 h

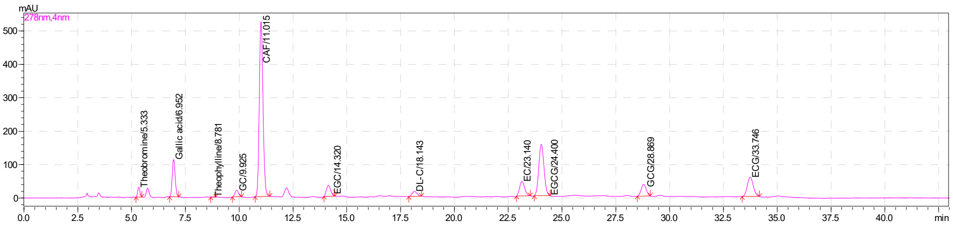

B-40 h

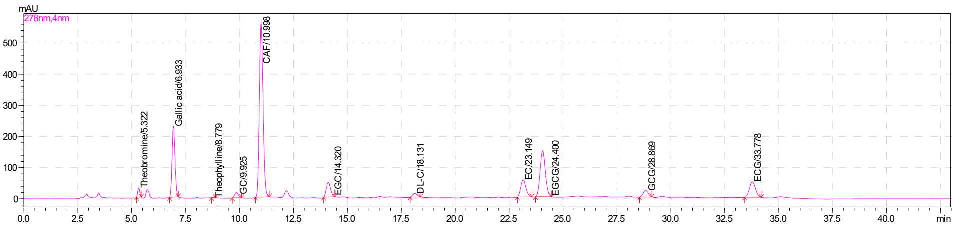

B-48 h

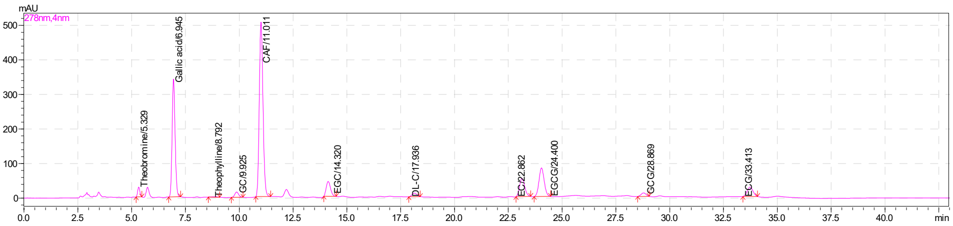

Figure S1 Standard and Sample Chromatograms: A: Standards. B: Samples (0h-48h).

The calibration curves and detailed information for each target compound are as follows：

Table S1. The calibration curves for each target compound

Theobromine	concentration（μg/mL）	25.2	45.1	65.1	85.1	105
	peak area	1321200	2378092	3595494	4659006	5877007
	Calibration	y=0.00002x+2.65635				
	R^2	0.9994				
Gallic acid	concentration（μg/mL）	20	40.1	59.5	80	99.5
	peak area	810926	1666007	2472006	3243706	4133688
	Calibration	y=0.00002x+0.22372				
	R^2	0.9993				
Theophylline	concentration（μg/mL）	15.5	25.2	35	45.3	55.5
	peak area	684147	1102434	1457948	1899292	2340004
	Calibration	y=0.00002x-1.12933				
	R^2	0.9992				
GC	concentration（μg/mL）	95	129	145	161	192
	peak area	328848	459766	519888	571789	684002
	Calibration	y=0.0003x+3.9055				
	R^2	0.9992				
Caffeine	concentration（μg/mL）	181	200	227	245	265
	peak area	8123368	9035935	10256454	11066714	12073340
	Calibration	y=0.00002x+6.66049				
	R^2	0.9996				
EGC	concentration（μg/mL）	195	210	234	255	265
	peak area	665780	719254	807187	874567	906350
	Calibration	y=0.0003x+1.446				
	R^2	0.9991				
DL-C	concentration（μg/mL）	95	145	207	225	250
	peak area	1161300	1767343	2524780	2752120	3009740
	Calibration	y=0.0001x-1.7618				

	R^2	0.9995				
EC	concentration（μg/mL）	95	155	216	230	269
	peak area	1378044	2082631	2904647	3103550	3616264
	Calibration	y=0.0001x-9.0253				
	R^2	0.9990				
EGCG	concentration（μg/mL）	83	100	123	150	174
	peak area	2206577	2596161	3193310	3895838	4566433
	Calibration	y=0.00004x-0.53333				
	R^2	0.9992				
GCG	concentration（μg/mL）	65	95	113	128	149
	peak area	1573630	2342145	2744705	3110666	3659607
	Calibration	y=0.00004x+0.9198				
	R^2	0.9994				
ECG	concentration（μg/mL）	84	100	155	174	216
	peak area	2631898	3125821	4953263	5535283	7033943
	Calibration	y=0.00003x+5.76031				
	R^2	0.9995				

Table S2. Dynamic changes of key chemical components during pile fermentation of dark raw tea

NO.	Physicochemical Indicators	0 h	8 h	16 h	24 h	32 h	40 h	48 h	P-ANOVA
1	Tea polyphenol (%)	18.21 ± 0.18	17.25 ± 0.23	17.30 ± 0.48	17.05 ± 0.12	17.01 ± 0.28	16.87 ± 0.33	16.82 ± 0.07	0.032
2	Gallic acid (mg/g)	0.028 ± 0.010	0.039 ± 0.010	0.050 ± 0.000	0.161 ± 0.019	0.416 ± 0.017	0.871 ± 0.019	1.388 ± 0.064	0.000
3	EGCG (mg/g)	2.524 ± 0.424	2.201 ± 0.270	2.481 ± 0.049	2.016 ± 0.121	1.782 ± 0.099	1.654 ± 0.020	1.177 ± 0.210	0.002
4	ECG (mg/g)	0.816 ± 0.127	0.771 ± 0.106	0.808 ± 0.026	0.705 ± 0.062	0.600 ± 0.017	0.505 ± 0.009	0.350 ± 0.072	0.002
5	EGC (mg/g)	2.763 ± 0.421	2.218 ± 0.232	2.503 ± 0.091	1.977 ± 0.095	2.271 ± 0.217	3.235 ± 0.051	3.409 ± 0.375	0.003
6	GCG (mg/g)	0.549 ± 0.077	0.554 ± 0.104	0.454 ± 0.089	0.444 ± 0.076	0.372 ± 0.060	0.266 ± 0.034	0.150 ± 0.033	0.001
7	GC (mg/g)	1.243 ± 0.101	1.214 ± 0.136	1.052 ± 0.142	0.933 ± 0.101	1.061 ± 0.059	1.054 ± 0.077	0.977 ± 0.084	0.042
8	EC (mg/g)	0.932 ± 0.132	0.854 ± 0.063	0.897 ± 0.017	0.777 ± 0.039	0.911 ± 0.079	1.149 ± 0.017	1.205 ± 0.092	0.005
9	DL-C (mg/g)	0.355 ± 0.051	0.349 ± 0.060	0.310 ± 0.058	0.294 ± 0.042	0.294 ± 0.042	0.277 ± 0.042	0.239 ± 0.025	0.079
10	Gallic acid (mg/g)	0.028 ± 0.010	0.039 ± 0.010	0.050 ± 0.000	0.161 ± 0.019	0.416 ± 0.017	0.871 ± 0.019	1.388 ± 0.064	0.000
11	Water extract (%)	40.51 ± 0.15	40.25 ± 0.53	38.49 ± 0.14	37.27 ± 1.28	37.19 ± 0.59	36.85 ± 0.13	36.10 ± 0.58	0.001
12	Free amino acid (%)	2.41 ± 0.12	2.17 ± 0.02	2.13 ± 0.02	2.06 ± 0.05	1.97 ± 0.01	2.03 ± 0.01	2.05 ± 0.06	0.001
13	Caffeine (mg/g)	2.236 ± 0.236	2.245 ± 0.145	2.359 ± 0.034	2.099 ± 0.126	2.271 ± 0.079	2.469 ± 0.026	2.387 ± 0.151	0.057
14	Theobromine (mg/g)	0.067 ± 0.00009	0.067 ± 0.00009	0.066 ± 0.00004	0.056 ± 0.00961	0.067 ± 0.00003	0.067 ± 0.00005	0.067 ± 0.00004	0.081
15	Crude protein (%)	18.058 ± 0.369	17.772 ± 0.044	15.946 ± 0.019	15.601 ± 0.219	15.359 ± 0.474	15.021 ± 0.143	14.367 ± 0.069	0.000
16	Soluble protein (mg/g)	33.263 ± 0.506	34.683 ± 1.038	34.190 ± 0.454	36.503 ± 0.241	37.723 ± 1.297	39.310 ± 0.366	41.743 ± 1.029	0.000
17	Protopectin (mg/g)	89.717 ± 8.433	86.027 ± 5.153	77.873 ± 1.769	71.593 ± 2.080	66.890 ± 2.185	63.293 ± 0.941	61.087 ± 5.091	0.002
18	Soluble pectin (mg/g)	20.763 ± 0.836	22.433 ± 0.484	23.627 ± 0.251	24.950 ± 0.440	25.203 ± 1.006	25.663 ± 0.291	26.647 ± 1.087	0.000
19	Cellulose (mg/g)	179.738±1.609	110.481±0.189	111.636±0.726	99.451±0.193	108.913±0.524	100.129±0.256	92.879±0.249	0.000
20	Soluble sugars (mg/g)	21.486±0.306	17.916±0.168	27.747±0.361	26.384±0.212	16.397±0.182	28.718±0.231	32.218±0.330	0.000
21	PPO (U/g)	144.167 ± 1.807	145.667 ± 14.370	155.967 ± 2.441	163.400 ± 3.647	165.900 ± 3.569	188.333 ± 4.955	203.233 ± 17.192	0.000

22	POD (U/g)	51.967 ± 3.035	53.933 ± 2.314	64.033 ± 5.117	74.833 ± 1.749	97.067 ± 3.059	126.500 ± 4.082	157.500 ± 6.864	0.000
23	Pectinase (U/g)	0.103 ± 0.011	0.103 ± 0.002	0.112 ± 0.003	0.225 ± 0.010	0.511 ± 0.032	1.735 ± 0.139	2.402 ± 0.216	0.000
24	Cellulase (U/g)	7.610 ± 0.686	7.383 ± 0.690	7.710 ± 0.550	11.040 ± 0.698	9.963 ± 0.455	9.797 ± 0.448	8.300 ± 0.676	0.023

Table S3. Information on 103 Aroma Compounds (μg/L)

NO.	Name	CAS	RI-Nist	RI-test	Category	0 h	8 h	16 h	24 h	32 h	40 h	48 h	*p*-Value	VIP
1	5-Ethyldecane	17302-36-2	1139	1137.4	Alkanes	0.92±0.17	1.4±0.19	1.5±0.14	1.62±0.12	1.87±0.46	1.86±0.18	1.72±0.26	0.011	0.82
2	4-Ethyldecane	1636-44-8	1141	1142.8	Alkanes	0±0	0±0	0±0	0±0	0.32±0.08	0.46±0.13	0.25±0.07	0.078	0.89
3	5-Methylundecane	1632-70-8	1156	1149.5	Alkanes	1.47±0.63	1.79±0.54	1.9±0.42	1.87±0.46	1.66±0.07	1.74±0.34	1.3±0.17	0.439	0.68
4	3-Methylundecane	1002-43-3	1170	1166	Alkanes	0±0	0±0	0±0	0±0	1.61±0.48	2.55±0.96	1.53±0.13	0.275	0.86
5	Dodecane	112-40-3	1200	1196.6	Alkanes	3.02±0.37	3.55±0.52	6.18±2	5.35±0.5	5.69±1.92	7.86±1.65	8.5±2.44	0.004	0.67
6	3,6-Dimethylundecane	17301-28-9	1210	1207.1	Alkanes	0±0	0±0	0±0	0±0	5.08±0.82	7.09±2.53	3.66±1.05	0.054	0.90
7	Tridecane	629-50-5	1300	1297.5	Alkanes	2.56±0.49	3.25±1.05	5.01±2.7	3.86±0.22	18.59±4.78	33.83±2.43	12.96±8.3	0.001	1.16
8	3-Methyltridecane	6418-41-3	1371	1367.7	Alkanes	0.94±0.25	0.89±0.18	1.02±0.28	1.17±0.39	6.99±1	7.72±4.71	4.48±2.88	0.002	0.83
9	3-Methylpentadecane	2882-96-4	1570	1564.9	Alkanes	0±0	0±0	0±0	0.93±0.15	1.76±0.07	1.71±0.47	1.34±0.15	0.024	0.95
10	Hexadecane	544-76-3	1600	1596.9	Alkanes	7.67±0.79	7.44±0.87	9.38±0.78	9.68±1.11	7.9±0.33	8.57±0.97	7.46±0.86	0.008	1.17
11	Heptadecane	629-78-7	1700	1697.4	Alkanes	9.75±1.18	9.05±1.25	13.97±2.73	13.23±1.34	10.58±0.72	9.52±0.77	8.9±1.33	0.004	1.09
12	Toluene	108-88-3	763	772.73	Aromatic hydrocarbons	98.06±8.01	97.19±10.01	99.89±10.35	89.19±2.6	87.47±2.8	102.05±10.83	119.41±21.16	0.047	1.11
13	1,3-Dimethylbenzene	108-38-3	866	864	Aromatic hydrocarbons	44.94±5.39	46.19±1.14	73.02±5.39	48.27±2.8	40.77±3.46	46.39±2.45	53.51±6.32	0.004	1.47
14	1,2-Dimethylbenzene	95-47-6	887	890.33	Aromatic hydrocarbons	0.63±0.33	1.05±0.87	2.27±1.01	1.62±0.37	7.05±0.31	7.55±1.29	7.81±0.38	0.001	0.83

15	1-Methyl-2-(1-methylethyl)-benzene	527-84-4	1022	1022.3	Aromatic hydrocarbons	12.07±0.5	12.12±0.89	12.36±0.63	12.75±1.01	9.75±0.74	9.73±0.9	12.75±1.24	0.006	1.17
16	1,6-Dimethylnaphthalene	575-43-9	1420	1417	Aromatic hydrocarbons	4.85±0.66	4.72±0.71	7.01±1.27	4.94±0.14	5.46±1.25	5.37±1.19	7.82±1.75	0.043	1.11
17	2-Pinene	80-56-8	937	925	Alkenes	36.93±4.35	41.57±2.28	50.13±3.59	58.69±5.37	30.64±2.26	32.97±4.32	27.47±2.06	0.001	1.23
18	Sabinene	3387-41-5	974	971.32	Alkenes	1.41±0.21	3.45±0.39	3.89±0.15	4.43±0.3	2.29±0.17	4.11±0.1	4.28±0.31	0.000	1.47
19	β-Myrcene	123-35-3	991	983.29	Alkenes	11.81±5.08	13.06±4.84	18.55±5.57	16.12±7.01	14.14±7.57	16.32±5.95	12.98±7.17	0.539	0.69
20	3-δ-Carene	13466-78-9	1011	1003.9	Alkenes	23.75±2.96	26.06±2.42	33.19±2.24	39.37±4.16	19.77±1.96	21.33±1.28	17.53±1.74	0.001	1.25
21	β-Ocimene	3338-55-4	1037	1042.5	Alkenes	42.17±5.48	51.2±6.05	46.74±1.84	36.26±2.6	36.95±2.7	35.67±3.35	31.82±3.87	0.003	1.15
22	γ-Terpinene	99-85-4	1060	1055	Alkenes	4.08±0.31	5.34±0.51	7.66±0.4	8.79±0.52	12.3±0.91	15.74±3.18	17.94±0.33	0.000	0.64
23	(3E)-4,8-Dimethyl-1,3,7-nonatriene	19945-61-0	1116	1110	Alkenes	1.46±0.14	2.13±0.43	1.79±0.13	1.8±0.2	1.75±0.23	1.68±0.16	1.71±0.1	0.053	1.28
24	α-Cubebene	17699-14-8	1351	1346	Alkenes	1.12±0.6	1.58±0.77	1.89±0.48	1.66±0.89	1.6±0.92	1.54±0.73	1.56±0.84	0.680	0.59
25	β-Cubebene	13744-15-5	1389	1387.3	Alkenes	0.92±0.22	1.21±0.4	1.28±0.32	1.27±0.46	1.07±0.53	1.02±0.27	1.25±0.25	0.715	0.68
26	α-Farnesene	502-61-4	1508	1501.3	Alkenes	12.21±2.96	12.97±5	14.07±0.94	10.66±0.46	15.88±4.24	13.49±3.45	14.11±1.16	0.247	1.05
27	Neophytadiene	504-96-1	1837	1834.7	Alkenes	28.8±8	31.21±5.71	40.25±8.94	34.68±8.61	35.72±10.18	30.65±2.84	37.56±9.71	0.543	0.85
28	1-Butanol	71-36-3	659	669.37	Alcohols	9.88±0.89	9.29±1.31	8.6±0.27	9.81±1.08	7.83±0.48	9.02±0.97	7.6±1.09	0.017	1.27
29	3-Methyl-1-butanol	123-51-3	736	731	Alcohols	32.91±1.73	27.67±1.43	35.18±2.15	30.11±5.1	31.63±2.58	33.36±6.9	33.04±2.43	0.105	1.26
30	1-Pentanol	71-41-0	765	754.07	Alcohols	0±0	0±0	4.98±0.15	7.71±0.68	28.37±3.06	34.34±6.08	27.69±8.94	0.003	0.75
31	(Z)-3-Hexenol	928-96-1	857	854	Alcohols	5.99±0.45	6.78±0.77	9.8±2.21	20.95±1.25	55.42±5.59	78.05±23.97	36.95±9.73	0.000	0.91
32	1-Hexanol	111-27-3	868	868.74	Alcohols	7.22±1.22	8.55±0.75	12.37±1.99	11.99±0.71	11.68±2.09	16.32±3.03	11.01±1.85	0.002	1.14
33	1-Octen-3-ol	3391-86-4	980	979.92	Alcohols	35.72±6.77	54.46±4.56	65.98±2.73	63.34±5.63	62.55±3.62	44.19±3.77	28.37±4.43	0.001	1.17
34	6-Methyl-5-hepten-2-ol	1569-60-4	994	994.09	Alcohols	5.18±0.54	9.13±0.27	13.96±1.78	14.15±3.09	22.96±1.61	26.19±3.52	27.01±1.44	0.000	0.68
35	Benzyl alcohol	100-51-6	1036	1048	Alcohols	0±0	0±0	0±0	7.45±0.31	8.56±1.2	10.24±0.5	12.26±1.22	0.005	0.96
36	cis-Linalool oxide	5989-33-3	1074	1067	Alcohols	24.53±2.54	36.75±2.06	46.64±1.05	50.24±4.77	63.15±7	69.1±5.81	72.28±1.24	0.000	0.69

	(furanoid)													
37	1-Octanol	111-87-5	1071	1073	Alcohols	16.95±1.2	23.48±2.05	30.75±1.95	27.8±2.24	24.93±1.07	25.02±2.21	19.93±1.18	0.001	1.04
38	trans-Linalool oxide	34995-77-2	1086	1084	Alcohols	21.48±2.4	35.02±1.91	46.24±1.22	52.42±4.93	66.29±7.52	75.66±7.83	80.52±3.07	0.000	0.67
39	Linalool	78-70-6	1099	1102.4	Alcohols	490.56±17.95	638.54±12.03	674.18±11.42	644.43±31.54	686.36±25.81	706.91±90.28	663.89±118.13	0.022	0.90
40	Phenethyl alcohol	60-12-8	1116	1121.2	Alcohols	17.26±0.75	32.93±6.56	54.19±5.52	83.49±17.05	116.3±11.78	181.39±34.31	181.68±7.27	0.000	0.68
41	(Z)-3-Nonen-1-ol	10340-23-5	1156	1156.2	Alcohols	8.74±1.74	10.15±0.7	12.51±0.71	12.37±1.57	11.21±1.1	8.25±1.17	6.76±0.39	0.001	1.07
42	(E)-2-Nonenol	31502-14-4	1176	1168	Alcohols	2.6±0.29	2.98±0.31	4.23±0.44	4.79±0.67	3.92±0.74	3.32±0.33	3.53±0.47	0.003	1.22
43	trans-Linalol oxide	39028-58-5	1174	1172	Alcohols	1±0.15	1.9±0.41	2.1±0.15	2.21±0.2	2.37±0.41	3.65±0.78	3.23±0.31	0.001	0.98
44	1-Nonanol	143-08-8	1173	1174.3	Alcohols	6.93±0.21	10.69±1.12	15.67±0.32	15.78±1.31	14.19±0.3	13.25±3.92	12.4±1.49	0.002	0.94
45	cis-Linalol oxide	14049-11-7	1178	1177.8	Alcohols	2.07±0.22	2.48±0.34	3.46±0.33	5.1±1.63	5.03±1.23	6.73±1.33	6.4±0.89	0.001	0.75
46	4-Terpineol	562-74-3	1177	1183.7	Alcohols	1.53±0.22	2.68±0.21	4.15±0.19	5.75±0.28	5.15±0.42	10.96±2.48	12.44±0.67	0.000	0.85
47	α-Terpinol	98-55-5	1189	1200.2	Alcohols	26.96±2.51	37.11±1.17	47.17±1.35	49.98±2.98	33.74±2.39	46.08±9.72	49.63±3.05	0.003	1.13
48	Nerol	106-25-2	1228	1226.3	Alcohols	34.69±2.15	50.01±7.92	64.99±1.03	47.47±5.27	33.96±5.03	25.11±2.11	24.34±2.33	0.000	0.95
49	Isogeraniol	5944-20-7	1240	1232.1	Alcohols	4.86±0.72	7.23±1.31	8.95±1.78	8.31±1.91	8.87±0.68	8.19±2.06	5.66±1.08	0.016	1.05
50	Geraniol	106-24-1	1255	1252.9	Alcohols	92.72±7.73	134.66±14.63	174.67±17.22	190.9±3.41	201.75±8.61	216.68±24.86	180.31±27.1	0.002	0.82
51	β-Ionol	22029-76-1	1428	1414.4	Alcohols	3.83±0.48	5.28±0.44	6.83±0.52	8.29±0.33	8.41±0.33	10.11±0.71	12.47±1.23	0.000	0.75
52	Epicubebol	38230-60-3	1493	1498.4	Alcohols	1.71±0.49	2.41±0.86	2.4±0.65	2.51±0.75	2.56±0.77	2.3±0.78	2.74±0.97	0.647	0.79
53	Cubebol	23445-02-5	1515	1519.2	Alcohols	1.45±0.26	2.36±0.12	3.09±0.34	2.77±0.15	2.54±0.36	2.48±0.2	3.27±0.63	0.003	0.94
54	Nerolidol	7212-44-4	1564	1562	Alcohols	140.39±12.63	148.06±18.96	164.14±13.26	166.23±9.39	195.27±19.04	179.08±24.63	196.08±22.9	0.007	0.98
55	Epicedrol	19903-73-2	1612	1617.2	Alcohols	2.8±0.37	2.47±0.39	2.5±0.08	2.63±0.22	2.27±0.18	2.6±0.1	3.01±0.39	0.078	1.00
56	Epicubenol	19912-67-5	1627	1631.9	Alcohols	1.34±0.15	2.01±0.05	2.21±0.21	1.92±0.27	1.87±0.16	2.18±0.44	3.26±0.32	0.003	1.02
57	α-Cadinol	481-34-5	1653	1663.2	Alcohols	3.56±0.25	4.09±0.39	3.56±0.24	3.61±0.18	2.9±0.21	2.5±0.28	3.91±0.73	0.004	1.19

58	Phytol	150-86-7	2114	2116.6	Alcohols	224.18±63.64	289.14±27.18	410.05±68.42	323.99±26.33	324.94±36.13	435.53±99.83	618.77±179	0.001	0.88
59	(Z)-4-Heptenal	6728-31-0	900	894.32	Aldehydes	2.17±0.14	3.39±0.46	5.12±0.59	5.62±0.84	7.5±0.82	8.98±0.63	9.62±1.55	0.000	0.63
60	Heptanal	111-71-7	901	897	Aldehydes	20.57±4.04	29.59±1.29	29.87±1.97	25.98±0.71	25.34±4.11	27.4±2.44	27.13±1.41	0.011	1.27
61	(E)-2-Heptenal	18829-55-5	958	953.61	Aldehydes	0±0	0±0	1.32±0.52	1.69±0.07	2.71±0.51	4.65±0.56	5.5±0.58	0.002	0.67
62	Benzaldehyde	100-52-7	962	963.05	Aldehydes	88.5±2.47	87.79±3.12	108.89±5.39	86.46±2.79	91.66±4.39	80.87±5.54	86.82±10.27	0.018	1.52
63	2,4-Heptadienal	5910-85-0	1012	995.76	Aldehydes	10.29±0.82	13.94±0.47	21.29±2.71	24.7±1.44	44.39±6.17	60.02±5.29	54.57±5.61	0.000	0.67
64	Octanal	124-13-0	1003	998.63	Aldehydes	9.4±1.61	10.35±0.26	10.6±0.43	9.01±0.91	8.04±0.44	7.24±0.77	7.24±0.58	0.004	0.79
65	(E,E)-2,4-Heptadienal	4313-03-5	1012	1013	Aldehydes	0±0	8.45±0.51	20.23±2.8	28.32±2.37	64.17±12.16	89.23±17.66	87.41±19.69	0.001	0.64
66	(E)-2-Octenal	2548-87-0	1060	1055.8	Aldehydes	0±0	1.64±0.23	3.38±0.74	4.85±0.57	7.46±0.79	12.1±1.39	11.94±2.16	0.001	0.67
67	Nonanal	124-19-6	1104	1102.4	Aldehydes	97.65±4.67	123.68±5.51	119.7±5.57	99.51±4.57	93.87±3.69	89.28±6.38	93.38±8.45	0.003	1.22
68	Decanal	112-31-2	1206	1204.3	Aldehydes	3.47±0.56	3.67±0.36	4.81±0.79	6.59±1.74	4.04±0.55	4.17±0.22	5.36±0.84	0.004	1.30
69	β-Cyclocitral	432-25-7	1220	1218.6	Aldehydes	7.87±0.72	8.7±0.3	11.1±0.33	11.76±0.61	12.44±0.78	15.14±3.15	15.77±2.17	0.001	0.66
70	β-Cyclohomocitral	472-66-2	1254	1254.6	Aldehydes	1.95±0.17	2.13±0.1	3.41±0.59	2.76±0.25	2.27±0.29	2.49±0.82	3.61±0.64	0.005	1.01
71	Geranial	141-27-5	1270	1267.9	Aldehydes	5.06±1.93	8.91±3.43	10.32±2.44	21.42±2.55	18.86±2.15	20.65±0.92	15.16±2.88	0.001	1.18
72	2-Heptanone	110-43-0	891	883.83	Ketones	0±0	1.53±0.14	2.01±0.16	2.22±0.47	3.72±0.6	2.98±0.41	2.59±0.13	0.001	1.08
73	6-Methyl-5-hepten-2-one	110-93-0	986	978.9	Ketones	11.05±1.48	13.83±0.85	20.73±1.97	21.71±1.85	26.09±2.23	26.31±2.73	27.47±2.24	0.001	0.75
74	2,2,6-Trimethylcyclohexanone	2408-37-9	1036	1031.1	Ketones	9.35±1.61	10.6±1.36	13.82±0.53	12.52±2.38	11.16±0.89	12.99±2.98	17.26±1.7	0.007	0.85
75	cis-Jasmone	488-10-8	1394	1393.7	Ketones	2.87±0.77	1.58±0.44	2.59±0.19	2.44±0.19	2.05±0.35	2.26±0.16	2.7±0.34	0.014	1.41
76	α-Ionone	127-41-3	1426	1420.6	Ketones	4.11±0.38	5.51±0.43	7.4±0.53	8.36±0.52	9.91±1.15	13.78±1.23	13.9±1.96	0.000	0.68
77	Geranylacetone	3796-70-1	1453	1444.7	Ketones	29.75±5	34.69±7.77	50.3±10.31	48.49±2.76	45.5±3.97	53.26±1.85	57.47±4.16	0.002	0.72
78	β-Ionone	79-77-6	1486	1477	Ketones	33.93±3.67	39.19±3.22	57.57±2.67	62.55±2.73	68.1±6.72	90.26±13.37	93.2±7.81	0.000	0.67
79	5,6-Epoxy-β-ionone	23267-57-4	1473	1479.8	Ketones	24.58±2.07	22.95±0.85	35.31±1.59	40.48±2.02	44.57±3.23	56.95±3.96	54.6±2.89	0.000	0.71

80	2-Tridecanone	593-08-8	1497	1492.8	Ketones	0.77±0.06	0.7±0.16	0.78±0.07	1.2±0.37	2.47±0.18	3.3±0.45	2.81±0.36	0.001	0.72
81	3-[(2E)-2-Pentenyl]-1,2,4-cyclopentanetrione	54644-27-8		1520.1	Ketones	74.58±5.54	76.56±5.55	157.83±6.21	144.42±10.48	111.71±7.57	119.43±6.52	137.91±14.87	0.000	1.11
82	(Z)-3-Hexenyl acetate	3681-71-8	1005	998.63	Esters	96.82±4.27	108.99±2.84	93.71±5	53.13±5.4	17.73±0.65	10.05±1.9	5.92±1.77	0.000	0.83
83	cis-3-Hexenyl butyrate	16491-36-4	1187	1180.3	Esters	12.4±0.91	20.2±1.69	19.72±0.87	15.69±0.89	11.65±0.85	10.28±0.47	7.36±0.93	0.000	1.04
84	Methyl salicylate	119-36-8	1192	1195.8	Esters	87.76±9.48	159.73±7.03	223.53±8.53	250.12±10.58	392.07±5.36	353.32±26.43	306.65±8.92	0.000	0.95
85	cis-3-Hexenyl-α-methyl butyrate	53398-85-9	1234	1225.4	Esters	5.45±0.45	6.84±0.43	7.2±0.43	5.85±0.17	5.34±0.24	5.47±0.66	5.19±0.76	0.006	1.17
86	cis-3-Hexenyl valerate	35852-46-1	1237	1238.4	Esters	10.98±0.64	12.39±0.3	14.74±0.74	14.01±0.55	13.27±0.39	12.33±1.6	12.11±1.82	0.006	0.95
87	L-bornyl acetate	5655-61-8	1284	1280.8	Esters	4.22±0.3	4.13±0.15	5.27±0.3	4.46±0.24	4.04±0.25	4.56±0.68	8.19±0.66	0.004	1.16
88	Methyl geranate	2349-14-6	1323	1319.8	Esters	12.23±1.62	15.04±1.74	18.33±0.81	19.19±0.78	20.32±1.36	20.56±1.08	20.97±2.06	0.001	0.74
89	(Z)-3-Hexenyl hexanoate	31501-11-8	1380	1376	Esters	6.39±0.72	6.49±0.63	7.46±0.86	6.3±0.26	6.78±0.76	6.74±1	6.93±0.7	0.496	0.98
90	cis-3-Hexenyl cis-3-hexenoate	61444-38-0	1389	1380	Esters	5.17±1.21	7.66±1.11	6.84±0.5	6.63±0.65	6.49±0.58	6.58±0.41	4.38±0.84	0.013	1.25
91	(Z)-3-Hexenyl (E)-2-hexenoate	53398-87-1		1430	Esters	1.91±0.17	2.21±0.31	1.78±0.04	1.86±0.18	1.78±0.19	1.68±0.35	1.54±0.55	0.126	0.99
92	Dihydroactinidiolide	17092-92-1	1532	1534	Esters	2.97±0.74	3.93±0.94	8.49±0.63	8.71±0.58	10.39±1.41	13.15±2.52	14.6±1.19	0.000	0.65
93	cis-3-Hexenyl benzoate	25152-85-6	1570	1573.7	Esters	3.23±0.74	4.4±0.62	6.14±1.08	5.31±0.43	4.85±0.83	4.31±0.57	3.99±0.59	0.008	0.94
94	Methyl jasmonate	1211-29-6	1638	1642	Esters	5.18±0.11	5.07±0.11	6.71±0.77	5.81±0.23	6.31±0.45	5.89±0.3	4.98±0.53	0.001	1.28
95	Methyl hexadecanoate	112-39-0	1926	1924.9	Esters	1.9±0.19	2.15±0.17	1.7±0.21	2±0.04	2.41±0.04	2.9±0.34	4.59±0.85	0.000	0.95
96	1,2,3-Trimethoxybenzene	634-36-6	1313	1307.6	Methoxy compounds	0±0	0±0	0±0	0±0	2.33±0.74	6.04±2.05	35.64±4.32	0.007	1.09
97	3,4-Dimethoxystyrene	6380-23-0	1369	1368.9	Methoxy compounds	0±0	0±0	0±0	2.15±0.28	6.05±0.21	8.82±1.62	9.18±0.97	0.006	0.67

98	Methyleugenol	93-15-2	1402	1401.4	Methoxy compounds	1.75±0.19	2.14±0.24	2.18±0.29	1.82±0.08	1.68±0.1	1.97±0.74	2.44±0.29	0.017	1.02
99	7-Methoxy-2,2-dimethyl chromene	17598-02-6	1466	1465.9	Methoxy compounds	90.15±5.29	185.22±17.99	117.25±6.57	112.71±5.19	108.82±7.73	107.87±9.08	193.2±29.51	0.001	1.59
100	2-Pentylfuran	3777-69-3	993	985.14	Others	10.93±2.01	11.08±2.41	5.81±0.35	5.62±0.84	10.77±2.7	10.75±6.72	9.95±1.22	0.019	0.92
101	Eucalyptol	470-82-6	1032	1026.6	Others	37.37±4.46	49.13±1.06	59.93±3.72	58.61±2.24	57.56±9.58	65.52±10.5	76.84±13.48	0.002	0.77
102	N,N-Dibutylformamide	761-65-9	1310	1300.3	Others	3.33±0.54	2.31±0.23	4.01±1.33	4.15±1.11	1.74±0.48	6.11±1.27	3.64±0.7	0.002	1.88
103	Diethyl Phthalate	84-66-2	1594	1586.5	Others	3.75±0.65	4.61±2.23	2.74±0.16	3.57±1.33	3.3±1.15	2.09±0.43	3.41±0.59	0.097	1.02

Table S4. List of identification information of the key differential substances (μg/L)

NO.	Name	CAS	RI-Nist	RI-test	Category	0 h	8 h	16 h	24 h	32 h	40 h	48 h	*p*-Value	VIP
1	Dodecane	112-40-3	1200	1196.6	Alkanes	3.02±0.37	3.55±0.52	6.18±2	5.35±0.5	5.69±1.92	7.86±1.65	8.5±2.44	0.004	0.67
2	Tridecane	629-50-5	1300	1297.5	Alkanes	2.56±0.49	3.25±1.05	5.01±2.7	3.86±0.22	18.59±4.78	33.83±2.43	12.96±8.3	0.001	1.16
3	3-Methyltridecane	6418-41-3	1371	1367.7	Alkanes	0.94±0.25	0.89±0.18	1.02±0.28	1.17±0.39	6.99±1	7.72±4.71	4.48±2.88	0.002	0.83
4	Hexadecane	544-76-3	1600	1596.9	Alkanes	7.67±0.79	7.44±0.87	9.38±0.78	9.68±1.11	7.9±0.33	8.57±0.97	7.46±0.86	0.008	1.17
5	Heptadecane	629-78-7	1700	1697.4	Alkanes	9.75±1.18	9.05±1.25	13.97±2.73	13.23±1.34	10.58±0.72	9.52±0.77	8.9±1.33	0.004	1.09
6	Toluene	108-88-3	763	772.73	Aromatic hydrocarbons	98.06±8.01	97.19±10.01	99.89±10.35	89.19±2.6	87.47±2.8	102.05±10.83	119.41±21.16	0.047	1.11
7	1,3-Dimethylbenzene	108-38-3	866	864	Aromatic hydrocarbons	44.94±5.39	46.19±1.14	73.02±5.39	48.27±2.8	40.77±3.46	46.39±2.45	53.51±6.32	0.004	1.47
8	1-Methyl-2-(1-methylethyl)-benzene	527-84-4	1022	1022.3	Aromatic hydrocarbons	12.07±0.5	12.12±0.89	12.36±0.63	12.75±1.01	9.75±0.74	9.73±0.9	12.75±1.24	0.006	1.17

9	1,6-Dimethylnaphthalene	575-43-9	1420	1417	Aromatic hydrocarbons	4.85±0.66	4.72±0.71	7.01±1.27	4.94±0.14	5.46±1.25	5.37±1.19	7.82±1.75	0.043	1.11
10	2-Pinene	80-56-8	937	925	Alkenes	36.93±4.35	41.57±2.28	50.13±3.59	58.69±5.37	30.64±2.26	32.97±4.32	27.47±2.06	0.001	1.23
11	Sabinene	3387-41-5	974	971.32	Alkenes	1.41±0.21	3.45±0.39	3.89±0.15	4.43±0.3	2.29±0.17	4.11±0.1	4.28±0.31	0.000	1.47
12	β-Myrcene	123-35-3	991	983.29	Alkenes	11.81±5.08	13.06±4.84	18.55±5.57	16.12±7.01	14.14±7.57	16.32±5.95	12.98±7.17	0.539	0.69
13	3-δ-Carene	13466-78-9	1011	1003.9	Alkenes	23.75±2.96	26.06±2.42	33.19±2.24	39.37±4.16	19.77±1.96	21.33±1.28	17.53±1.74	0.001	1.25
14	β-Ocimene	3338-55-4	1037	1042.5	Alkenes	42.17±5.48	51.2±6.05	46.74±1.84	36.26±2.6	36.95±2.7	35.67±3.35	31.82±3.87	0.003	1.15
15	(3E)-4,8-Dimethyl-1,3,7-nonatriene	19945-61-0	1116	1110	Alkenes	1.46±0.14	2.13±0.43	1.79±0.13	1.8±0.2	1.75±0.23	1.68±0.16	1.71±0.1	0.053	1.28
16	α-Farnesene	502-61-4	1508	1501.3	Alkenes	12.21±2.96	12.97±5	14.07±0.94	10.66±0.46	15.88±4.24	13.49±3.45	14.11±1.16	0.247	1.05
17	Neophytadiene	504-96-1	1837	1834.7	Alkenes	28.8±8	31.21±5.71	40.25±8.94	34.68±8.61	35.72±10.18	30.65±2.84	37.56±9.71	0.543	0.85
18	1-Butanol	71-36-3	659	669.37	Alcohols	9.88±0.89	9.29±1.31	8.6±0.27	9.81±1.08	7.83±0.48	9.02±0.97	7.6±1.09	0.017	1.27
19	3-Methyl-1-butanol	123-51-3	736	731	Alcohols	32.91±1.73	27.67±1.43	35.18±2.15	30.11±5.1	31.63±2.58	33.36±6.9	33.04±2.43	0.105	1.26
20	(Z)-3-Hexenol	928-96-1	857	854	Alcohols	5.99±0.45	6.78±0.77	9.8±2.21	20.95±1.25	55.42±5.59	78.05±23.97	36.95±9.73	0.000	0.91
21	1-Hexanol	111-27-3	868	868.74	Alcohols	7.22±1.22	8.55±0.75	12.37±1.99	11.99±0.71	11.68±2.09	16.32±3.03	11.01±1.85	0.002	1.14
22	1-Octen-3-ol	3391-86-4	980	979.92	Alcohols	35.72±6.77	54.46±4.56	65.98±2.73	63.34±5.63	62.55±3.62	44.19±3.77	28.37±4.43	0.001	1.17
23	1-Octanol	111-87-5	1071	1073	Alcohols	16.95±1.2	23.48±2.05	30.75±1.95	27.8±2.24	24.93±1.07	25.02±2.21	19.93±1.18	0.001	1.04
24	Linalool	78-70-6	1099	1102.4	Alcohols	490.56±17.95	638.54±12.03	674.18±11.42	644.43±31.54	686.36±25.81	706.91±90.28	663.89±118.13	0.022	0.90
25	Phenethyl alcohol	60-12-8	1116	1121.2	Alcohols	17.26±0.75	32.93±6.56	54.19±5.52	83.49±17.05	116.3±11.78	181.39±34.31	181.68±7.27	0.000	0.68
26	(Z)-3-Nonen-1-ol	10340-23-5	1156	1156.2	Alcohols	8.74±1.74	10.15±0.7	12.51±0.71	12.37±1.57	11.21±1.1	8.25±1.17	6.76±0.39	0.001	1.07
27	(E)-2-Nonenol	31502-14-4	1176	1168	Alcohols	2.6±0.29	2.98±0.31	4.23±0.44	4.79±0.67	3.92±0.74	3.32±0.33	3.53±0.47	0.003	1.22
28	α-Terpinol	98-55-5	1189	1200.2	Alcohols	26.96±2.51	37.11±1.17	47.17±1.35	49.98±2.98	33.74±2.39	46.08±9.72	49.63±3.05	0.003	1.13
29	Isogeraniol	5944-20-7	1240	1232.1	Alcohols	4.86±0.72	7.23±1.31	8.95±1.78	8.31±1.91	8.87±0.68	8.19±2.06	5.66±1.08	0.016	1.05

30	Geraniol	106-24-1	1255	1252.9	Alcohols	92.72±7.73	134.66±14.63	174.67±17.22	190.9±3.41	201.75±8.61	216.68±24.86	180.31±27.1	0.002	0.82
31	Nerolidol	7212-44-4	1564	1562	Alcohols	140.39±12.63	148.06±18.96	164.14±13.26	166.23±9.39	195.27±19.04	179.08±24.63	196.08±22.9	0.007	0.98
32	Epicedrol	19903-73-2	1612	1617.2	Alcohols	2.8±0.37	2.47±0.39	2.5±0.08	2.63±0.22	2.27±0.18	2.6±0.1	3.01±0.39	0.078	1.00
33	Epicubenol	19912-67-5	1627	1631.9	Alcohols	1.34±0.15	2.01±0.05	2.21±0.21	1.92±0.27	1.87±0.16	2.18±0.44	3.26±0.32	0.003	1.02
34	α-Cadinol	481-34-5	1653	1663.2	Alcohols	3.56±0.25	4.09±0.39	3.56±0.24	3.61±0.18	2.9±0.21	2.5±0.28	3.91±0.73	0.004	1.19
35	Phytol	150-86-7	2114	2116.6	Alcohols	224.18±63.64	289.14±27.18	410.05±68.42	323.99±26.33	324.94±36.13	435.53±99.83	618.77±179	0.001	0.88
36	Heptanal	111-71-7	901	897	Aldehydes	20.57±4.04	29.59±1.29	29.87±1.97	25.98±0.71	25.34±4.11	27.4±2.44	27.13±1.41	0.011	1.27
37	Benzaldehyde	100-52-7	962	963.05	Aldehydes	88.5±2.47	87.79±3.12	108.89±5.39	86.46±2.79	91.66±4.39	80.87±5.54	86.82±10.27	0.018	1.52
38	(E,E)-2,4-Heptadienal	4313-03-5	1012	1013	Aldehydes	0±0	8.45±0.51	20.23±2.8	28.32±2.37	64.17±12.16	89.23±17.66	87.41±19.69	0.001	0.64
39	(E)-2-Octenal	2548-87-0	1060	1055.8	Aldehydes	0±0	1.64±0.23	3.38±0.74	4.85±0.57	7.46±0.79	12.1±1.39	11.94±2.16	0.001	0.67
40	Nonanal	124-19-6	1104	1102.4	Aldehydes	97.65±4.67	123.68±5.51	119.7±5.57	99.51±4.57	93.87±3.69	89.28±6.38	93.38±8.45	0.003	1.22
41	Decanal	112-31-2	1206	1204.3	Aldehydes	3.47±0.56	3.67±0.36	4.81±0.79	6.59±1.74	4.04±0.55	4.17±0.22	5.36±0.84	0.004	1.30
42	β-Cyclohomocitral	472-66-2	1254	1254.6	Aldehydes	1.95±0.17	2.13±0.1	3.41±0.59	2.76±0.25	2.27±0.29	2.49±0.82	3.61±0.64	0.005	1.01
43	Geranial	141-27-5	1270	1267.9	Aldehydes	5.06±1.93	8.91±3.43	10.32±2.44	21.42±2.55	18.86±2.15	20.65±0.92	15.16±2.88	0.001	1.18
44	2-Heptanone	110-43-0	891	883.83	Ketones	0±0	1.53±0.14	2.01±0.16	2.22±0.47	3.72±0.6	2.98±0.41	2.59±0.13	0.001	1.08
45	cis-Jasmone	488-10-8	1394	1393.7	Ketones	2.87±0.77	1.58±0.44	2.59±0.19	2.44±0.19	2.05±0.35	2.26±0.16	2.7±0.34	0.014	1.41
46	β-Ionone	79-77-6	1486	1477	Ketones	33.93±3.67	39.19±3.22	57.57±2.67	62.55±2.73	68.1±6.72	90.26±13.37	93.2±7.81	0.000	0.67
47	5,6-Epoxy-β-ionone	23267-57-4	1473	1479.8	Ketones	24.58±2.07	22.95±0.85	35.31±1.59	40.48±2.02	44.57±3.23	56.95±3.96	54.6±2.89	0.000	0.71
48	3-[(2E)-2-Pentenyl]-1,2,4-cyclopentanetrione	54644-27-8		1520.1	Ketones	74.58±5.54	76.56±5.55	157.83±6.21	144.42±10.48	111.71±7.57	119.43±6.52	137.91±14.87	0.000	1.11
49	cis-3-Hexenyl butyrate	16491-36-4	1187	1180.3	Esters	12.4±0.91	20.2±1.69	19.72±0.87	15.69±0.89	11.65±0.85	10.28±0.47	7.36±0.93	0.000	1.04
50	cis-3-Hexenyl-α-methyl	53398-85-9	1234	1225.4	Esters	5.45±0.45	6.84±0.43	7.2±0.43	5.85±0.17	5.34±0.24	5.47±0.66	5.19±0.76	0.006	1.17

	butyrate													
51	L-bornyl acetate	5655-61-8	1284	1280.8	Esters	4.22±0.3	4.13±0.15	5.27±0.3	4.46±0.24	4.04±0.25	4.56±0.68	8.19±0.66	0.004	1.16
52	cis-3-Hexenyl cis-3-hexenoate	61444-38-0	1389	1380	Esters	5.17±1.21	7.66±1.11	6.84±0.5	6.63±0.65	6.49±0.58	6.58±0.41	4.38±0.84	0.013	1.25
53	Dihydroactinidiolide	17092-92-1	1532	1534	Esters	2.97±0.74	3.93±0.94	8.49±0.63	8.71±0.58	10.39±1.41	13.15±2.52	14.6±1.19	0.000	0.65
54	Methyl jasmonate	1211-29-6	1638	1642	Esters	5.18±0.11	5.07±0.11	6.71±0.77	5.81±0.23	6.31±0.45	5.89±0.3	4.98±0.53	0.001	1.28
55	1,2,3-Trimethoxybenzene	634-36-6	1313	1307.6	Methoxy compounds	0±0	0±0	0±0	0±0	2.33±0.74	6.04±2.05	35.64±4.32	0.007	1.09
56	Methyleugenol	93-15-2	1402	1401.4	Methoxy compounds	1.75±0.19	2.14±0.24	2.18±0.29	1.82±0.08	1.68±0.1	1.97±0.74	2.44±0.29	0.017	1.02
57	7-Methoxy-2,2-dimethylchromene	17598-02-6	1466	1465.9	Methoxy compounds	90.15±5.29	185.22±17.99	117.25±6.57	112.71±5.19	108.82±7.73	107.87±9.08	193.2±29.51	0.001	1.59
58	N,N-Dibutylformamide	761-65-9	1310	1300.3	Others	3.33±0.54	2.31±0.23	4.01±1.33	4.15±1.11	1.74±0.48	6.11±1.27	3.64±0.7	0.002	1.88
59	Diethyl Phthalate	84-66-2	1594	1586.5	Others	3.75±0.65	4.61±2.23	2.74±0.16	3.57±1.33	3.3±1.15	2.09±0.43	3.41±0.59	0.097	1.02
